# Impaired Autophagy in CD11b^+^ Dendritic Cells Expands CD4^+^ Regulatory T Cells and Limits Atherosclerosis in Mice

**DOI:** 10.1161/CIRCRESAHA.119.315248

**Published:** 2019-11-07

**Authors:** Marc Clement, Juliette Raffort, Fabien Lareyre, Dimitrios Tsiantoulas, Stephen Newland, Yuning Lu, Leanne Masters, James Harrison, Svetlana Saveljeva, Marcella K.L. Ma, Maria Ozsvar-Kozma, Brian Y.H. Lam, Giles S.H. Yeo, Christoph J. Binder, Arthur Kaser, Ziad Mallat

**Affiliations:** 1 From the Division of Cardiovascular Medicine, University of Cambridge, Cambridge, United Kingdom (M.C., J.R., F.L., D.T., S.N., Y.L., L.M., J.H., Z.M.); 2 Université CÔte d’Azur, Institut National de la Santé et de la Recherche Médicale, Centre Mediterranéen de Recherche Moléculaire, University Hospital of Nice, France (J.R., F.L.); 3 Department of Gastroenterology and Hepatology, University of Cambridge, United Kingdom (S.S., A.K.); 4 MRC Metabolic Diseases Unit, University of Cambridge Metabolic Research Laboratories, Wellcome Trust-MRC Institute of Metabolic Science, Genomics and Transcriptomics Core, Addenbrooke’s Hospital, Cambridge, United Kingdom (M.K.L.M., B.Y.H.L., G.S.H.Y.); 5 Department of Laboratory Medicine, Medical University of Vienna and Center for Molecular Medicine (CeMM) of the Austrian Academy of Sciences Vienna, Austria (M.O.-K., C.J.B); 6 Institut National de la Santé et de la Recherche Médicale, Paris Cardiovascular Research Center, France (Z.M.).

**Keywords:** atherosclerosis, autophagy, dendritic cells, immune system, macrophages

## Abstract

Atherosclerosis is a chronic inflammatory disease. Recent studies have shown that dysfunctional autophagy in endothelial cells, smooth muscle cells, and macrophages, plays a detrimental role during atherogenesis, leading to the suggestion that autophagy-stimulating approaches may provide benefit.

**Objective:**

Dendritic cells (DCs) are at the crossroad of innate and adaptive immune responses and profoundly modulate the development of atherosclerosis. Intriguingly, the role of autophagy in DC function during atherosclerosis and how the autophagy process would impact disease development has not been addressed.

**Methods and Results:**

Here, we show that the autophagic flux in atherosclerosis-susceptible *Ldlr*^−/−^ (low-density lipoprotein receptor–deficient) mice is substantially higher in splenic and aortic DCs compared with macrophages and is further activated under hypercholesterolemic conditions. RNA sequencing and functional studies on selective cell populations reveal that disruption of autophagy through deletion of *Atg16l1* differentially affects the biology and functions of DC subsets in *Ldlr*^−/−^ mice under high-fat diet. *Atg16l1* deficient CD11b^+^ DCs develop a TGF (transforming growth factor)-β–dependent tolerogenic phenotype and promote the expansion of regulatory T cells, whereas no such effects are seen with *Atg16l1* deficient CD8α^+^ DCs. *Atg16l1* deletion in DCs (all CD11c-expressing cells) expands aortic regulatory T cells in vivo, limits the accumulation of T helper cells type 1, and reduces the development of atherosclerosis in *Ldlr*^−/−^ mice. In contrast, no such effects are seen when *Atg16l1* is deleted selectively in conventional CD8α^+^ DCs and CD103^+^ DCs. Total T-cell or selective regulatory T-cell depletion abrogates the atheroprotective effect of *Atg16l1* deficient DCs.

**Conclusions:**

In contrast to its proatherogenic role in macrophages, autophagy disruption in DCs induces a counter-regulatory response that maintains immune homeostasis in *Ldlr*^−/−^ mice under high-fat diet and limits atherogenesis. Selective modulation of autophagy in DCs could constitute an interesting therapeutic target in atherosclerosis.

## Abstract 2

Supplemental Digital Content is available in the text.

Extensive basic, preclinical and translational studies have validated the inflammatory hypothesis of atherosclerosis.^1^ This concept has recently been nicely supported by the results of the CANTOS trial (Canakinumab Antiinflammatory Thrombosis Outcome Study), which showed a significant reduction of cardiovascular events in patients with stable coronary artery disease and residual inflammation after treatment with canakinumab, a monoclonal anti–IL (interleukin) 1β antibody.^2^ However, the relatively limited size effect of canakinumab and the failure of other anti-inflammatory therapies to alter the disease process in humans^3^ crucially highlight the importance of a better understanding of the complex regulation of the immune system in the context of atherosclerosis.

Autophagy has recently emerged as a major modulator of a variety of cellular functions with high relevance to the development and progression of atherosclerosis.^4^ Dysfunctional autophagy in atherosclerosis promotes apoptosis and senescence of endothelial cells,^5^ premature senescence of vascular smooth muscle cells (SMCs),^6^ disturbs the cholesterol efflux pathway^7^ and activates NLRP3 (NOD [nucleotide-binding oligomerization domain]-like receptor family, pyrin domain containing 3) inflammasome in macrophages,^8^ and impairs the efferocytosis of apoptotic cells,^9^ all processes involved in plaque inflammation, progression and complications.

The relevance of autophagy in cells of the adaptive immune system to the development of atherosclerosis has received little attention. A recent study reported decreased atherosclerosis in mice with autophagy-related protein (*Atg7*) deletion in T cells.^10^ The atheroprotective effect could not be attributed to a reduction of T-cell–mediated inflammation because *Atg7*-deficient T cells produced higher levels of the proatherogenic IFN (interferon)-γ. Htowever, *Atg7* deficiency in T cells was associated with an unexplained reduction of plasma cholesterol levels, which may have accounted for the atheroprotective effects. Given that dysfunctional autophagy may impair T helper cell differentiation, effector cell activation^11^ and anergy,^12^ memory formation,^13^ as well as regulatory T-cell (Treg) responses,^14^ addressing the role of autophagy in selective T-cell subsets is necessary for a better understanding of the relevance of those processes to atherogenesis.

Dendritic cells (DCs) are professional antigen-presenting cells at the crossroad of innate and adaptive immune responses. DCs originate from a DC progenitor in the bone marrow. Transcription factors influencing DC subset development include Zbtb46 (zinc finger and BTB domain containing 46) for preclassical DCs, which also require BATF3 (basic leucine zipper activating transcription factor–like transcription factor 3) and IRF8 (IFN regulatory factor) to differentiate into CD103^+^ (CD8α^+^ in lymphoid tissue) conventional DCs (cDCs) or RBPJ (recombination signal binding protein for immunoglobulin kappa J) and IRF4 to give rise to CD11b^+^ cDCs. In contrast, E2-2 (TCF4 [transcription factor 4]) is required for differentiation of the DC progenitor into plasmacytoid DCs. DC subsets may promote or limit atherogenesis through modulation of both innate and adaptive immune responses.^15,16^

Although it is dispensable for DC development, autophagy is involved in several biological processes relevant to DC functions, including DC maturation, responses to toll-like receptor stimulation, and cytokine production, migration, antigen presentation and cross-presentation, and T-cell activation (reviewed in Ghislat and Lawrence^17^). DCs profoundly alter the development of atherosclerosis through effects on lipid metabolism, T-cell priming, activation and differentiation, and modulation of Treg responses.^15,18–20^ Intriguingly, however, no study has addressed the role of autophagy in modulating DC functions during the development of atherosclerosis. Here, we aimed to fill this gap of knowledge and examined the impact of dysfunctional autophagy in distinct DC subsets on the immune responses during atherosclerosis. To modulate autophagy in DCs, we have deleted ATG16L1, which binds ATG5 and links the isolation membrane to the formation of the autophagosome.^21,22^

## Methods

Detailed methods are described in the Online Data Supplement.

All the experiments were approved by the local ethics committee and were performed under Home Office, UK license PA4BDF775. All the mice were on a C57Bl/6J genetic background. Female *Ldlr*^−/−^ (low-density lipoprotein receptor–deficient) mice (6–8 week-old) were lethally irradiated (9.5 Gy), then injected intravenously (tail vein) with 1×10^7^ bone marrow cells from donor mice. After 4 weeks of recovery, mice were fed a chow or high-fat diet (HFD; 21 % fat, 0.15 % cholesterol; Special Diet Services) for 8 weeks. Female littermate *CD11c*^*Cre*−^ *Atg16l1*^*flox/flox*^ and *CD11c*^*Cre+*^ *Atg16l1*^*flox/flox*^, as well as female littermate C-type lectin domain containing 9A (*Clec9a*)^*Cre−*^ *Atg16l*^*flox/flox*^ and *Clec9a*^*Cre+*^ *Atg16l1*^*flox/flox*^, were used as bone marrow donors to reconstitute lethally irradiated *Ldlr*^−/−^ animals.

## Results

### HFD Induces Autophagy in Aortic CD103^+^ and Splenic CD11b^+^ DCs in Ldlr^−/−^ Mice

**Figure 1.**
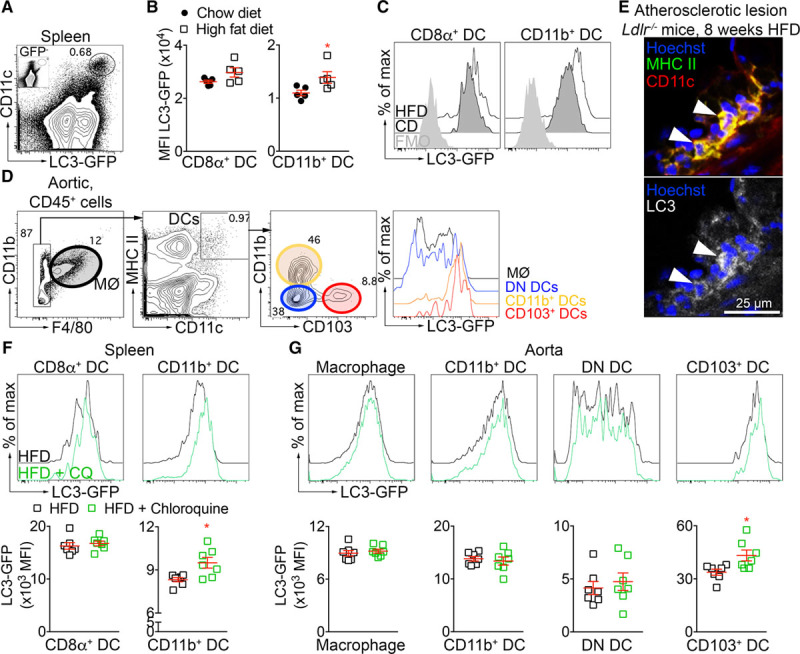
**Activation of the autophagic flux in dendritic cells under atherogenic conditions**. **A**, Representative flow chart showing expression of LC3 (microtubule-associated protein 1A/1B-light chain 3)-GFP (green fluorescent protein) in CD11c^high^ cells in the spleen of *Ldlr*^−/−^ (low-density lipoprotein receptor–deficient) mice transplanted with LC3-GFP bone marrow and put under high-fat diet (HFD) for 8 wk. The inset represents the same staining from a wild-type mouse (not expressing the LC3-GFP). **B**, Quantification of LC3-GFP mean fluorescence intensity (MFI) on CD11b^+^ dendritic cells (DCs) in the spleen of *Ldlr*^−/−^ mice transplanted with LC3-GFP bone marrow and left on chow diet (CD) or put under HFD for 8 wk. *P=0.0317 CD11b^+^ DCs chow diet vs CD11b^+^ DCs HFD, Mann-Whitney test. **C**, Representative histogram of flow cytometry showing the expression of LC3-GFP in CD8α^+^ and CD11b^+^ DC under CD or HFD. **D**, Flow charts depicting the gating strategy to analyze macrophages (CD45^+^CD11b^+^F4/80^+^) and DCs (CD45^+^F4/80^−^CD11c^+^MHC [major histocompatibility complex] II^high^) subsets (CD11b^+^ DCs [CD11b^+^CD103^−^], double negative (DN) DCs [CD11b^−^CD103^−^], CD103^+^ DCs [CD11b^−^CD103^+^]) as well as their expression of LC3-GFP, from the aorta of *Ldlr*^−/−^ mice transplanted with LC3-GFP and kept under HFD for 8 wk. **A**–**D**, n=5 mice/group. **E**, Representative confocal images of DCs (CD11c^+^MHCII^+^, white arrows) and LC3 staining in atherosclerotic lesions from the aortic root of *Ldlr*^−/−^ mice kept under HFD for 8 wk. **F**, Representative flow histograms and quantification of the expression of LC3-GFP in splenic DC subsets (CD8α^+^ and CD11b^+^ DCs) of *Ldlr*^−/−^ mice transplanted with LC3-GFP bone marrow and put on HFD for 8 wk. Chloroquine (CQ) was injected intraperitoneally 48 and 24 h before the end of the experiment to prevent the degradation of GFP by lysosomes. *P=0.0379 CD11b^+^ DCs HFD vs CD11b^+^ DCs HFD+CQ, Mann-Whitney test. **G**, Representative flow histograms and quantification of the expression of LC3-GFP in aortic macrophages and DC subsets (CD11b^+^, DN and CD103^+^ DCs) of *Ldlr*^−/−^ mice transplanted with LC3-GFP bone marrow and put on HFD for 8 wk. CQ was administered as in **F**. **F** and **G**: n=7 mice/group, *P=0.0216 CD103^+^ DCs HFD vs CD103^+^ DCs HFD+CQ, Mann-Whitney test, data were obtained from 1 experiment. FMO indicates fluorescence minus one control.

Using bone marrow transfer from LC3 (microtubule-associated protein 1A/1B-light chain 3)-GFP (green fluorescent protein) mice^23^ to *Ldlr*^−/−^ mice, we first studied the modification of the autophagic flux in DCs after long term HFD feeding. In the spleen, DCs and more particularly cDCs express high levels of LC3-GFP (Figure 1A) compared with monocytes, B cells, or T cells (Online Figure IA and IB), and this may be further enhanced by the HFD in CD11b^+^ DCs (Figure 1B and 1C). In the aorta, CD103^+^ DCs express the highest levels of LC3-GFP, followed by CD11b^+^ DCs, CD11b^−^CD103^−^ DCs, and macrophages (Figure 1D and Online Figure IC). HFD feeding did not change LC3-GFP expression in aortic DCs and macrophages (Online Figure IC). We confirmed by immunostaining that CD11c^+^MHC (major histocompatibility complex) II^+^LC3^+^ cells were found in the atherosclerotic plaque (Figure 1E). To know if the autophagic flux was active in DCs after HFD feeding, we used chloroquine injection in vivo to inhibit autophagosome fusion with lysosomes and limit the degradation of LC3-GFP. LC3-GFP expression in splenic CD11b^+^ DCs increased in the presence of chloroquine, suggesting that this was an active process (Figure 1F). However, chloroquine did not affect LC3-GFP expression in splenic CD8α^+^ DCs. In the aorta, chloroquine significantly enhanced the expression of LC3-GFP in CD103^+^ DCs, whereas no changes were observed in the other subsets (Figure 1G). Although a short-term injection of chloroquine can only inform about the activity of the autophagic flux at a specific time point (during the 2 days between chloroquine injection and mouse sacrifice), the data indicate that the autophagic flux is active both in splenic and aortic DCs under HFD.

### Atg16l1 Deficiency in CD11c-Expressing Cells Alters Splenic T-Cell Expansion and Promotes CD4^+^ Tregs in Ldlr^−/−^ Mice After HFD

**Figure 2.**
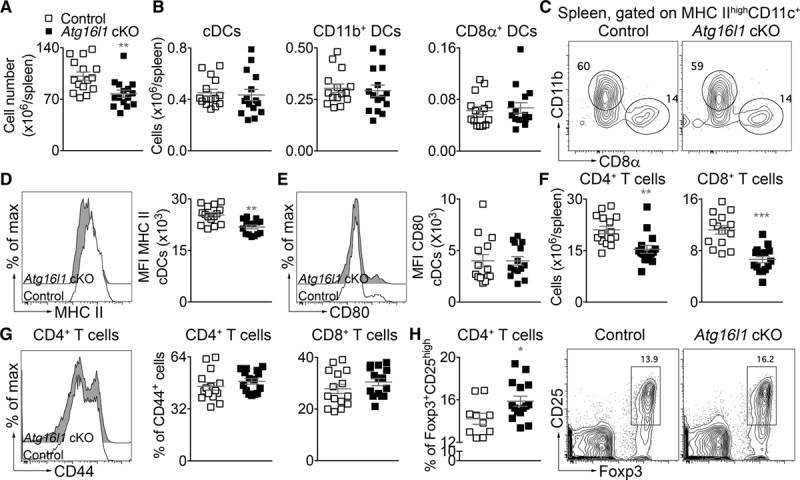
**Atg16l1 deficiency in CD11c-expressing cells promotes immune tolerance under high-fat diet (HFD) conditions in Ldlr−/− (low-density lipoprotein receptor–deficient) mice**. **A**, Absolute number of immune cells in the spleen of control (*Ldlr*^−/−^ mice transplanted with *CD11c*^*Cre−*^ *Atg16l1*^*flox/flox*^ bone marrow) and *Atg16l1* conditional knock out (cKO; *Ldlr*^−/−^ mice transplanted with *CD11c*^*Cre+*^ *Atg16l1*^*flox/flox*^ bone marrow) mice after 8 wk of HFD. n=15 mice/group. **P=0.0017 control vs *Atg16l1* cKO, Mann-Whitney test. **B**, Absolute number of conventional dendritic cells (cDCs), CD11b^+^, and CD8α^+^ DCs in the spleen of control (n=10) and *Atg16l1* cKO mice (n=15) after 8 wk of HFD, after flow cytometric analysis. **C**, Representative flow chart showing the distribution of CD8α^+^ and CD11b^+^ DC subsets in the spleen of control and *Atg16l1* cKO mice after 8 wk of HFD. **D**, Representative flow chart and quantification of MHC (major histocompatibility complex) II expression by cDCs in the spleen of control (n=10) and *Atg16l1* cKO mice (n=15) after 8 wk of HFD. **P=0.0014 control DC vs *Atg16l1* cKO DC, Mann-Whitney test. **E**, Representative flow chart and quantification of CD80 expression by cDCs in the spleen of control (n=10) and *Atg16l1* cKO (n=15) mice after 8 wk of HFD. **F**, Absolute number of CD4^+^ and CD8^+^ T cells in the spleen of control (n=10) and *Atg16l1* cKO mice (n=15) after 8 wk of HFD, after flow cytometric analysis. ***P=0.0006 number of splenic CD4^+^ T cells and ***P<0.0001 number of splenic CD8^+^ T cells in control vs *Atg16l1* cKO mice, Mann-Whitney test. **G**, Representative flow chart of CD44 expression by CD4^+^ T cells and quantification of the percentage of CD4^+^ and CD8^+^ T cells expressing CD44 in the spleen of control (n=10) and *Atg16l1* cKO mice (n=15) after 8 wk of HFD. **H**, Quantification and representative flow charts showing the proportion of CD4^+^ regulatory T cells (Tregs; Foxp3 [forkhead box P]^+^CD25^high^) in the spleen of control (n=10) and *Atg16l1* cKO mice (n=15) after 8 wk of HFD. *P=0.0137, %CD4^+^ Tregs in control vs *Atg16l1* cKO mice, Mann-Whitney test. **A**–**H**, Results are representative of 3 independent experiments.

To examine whether autophagy in DCs plays a role in atherosclerosis, bone marrow from *Atg16l1*^*flox/flox*^ *Cd11c*^*Cre+*^ (designated thereafter as *Atg16l1* conditional knock out [cKO]) or *Cd11c*^*Cre−*^ (designated thereafter as controls) littermate mice was transferred into *Ldlr*^−/−^ recipient mice. After 4 weeks of recovery, mice were fed a HFD for 8 weeks. At euthanization, plasma total cholesterol, HDL (high-density lipoprotein)-cholesterol, triglycerides, and weight were not different between the 2 groups (Online Figure II). Phenotyping of the spleen by flow cytometry revealed a significant reduction of spleen cellularity in *Atg16l1* cKO as compared to control *Ldlr*^−/−^ mice (Figure 2A). No differences in the numbers of DC subsets were observed between the 2 groups (Figure 2B-C). However, DCs of *Atg16l1* cKO *Ldlr*^−/−^ mice showed a reduction in MHCII expression (Figure 2D) without differences in CD80 (Figure 2E), as compared to DC from control *Ldlr*^−/−^ mice. Interestingly, deletion of *Atg16l1* in DCs was associated with a reduction of the numbers of splenic CD8^+^ and CD4^+^ T cells (Figure 2F), without affecting their expression of CD44 (memory T cells; Figure 2G). In parallel, we found that the proportion of CD4^+^ Tregs was increased in *Atg16l1* cKO compared with control *Ldlr*^−/−^ mice (Figure 2H). B cells, germinal center B cells, and T follicular helper cells, as well as immunoglobulin production (total and oxidized lipoprotein-specific), were not statistically different between the 2 groups (Online Figure III). Other splenic myeloid populations, including neutrophils and monocytes/macrophages, were likewise similar (*P*>0.05) between the 2 groups (Online Figure IV). Importantly, we confirmed that the DC and T-cell phenotype described above was not observed under normocholesterolemic conditions (Online Figure V), suggesting a role for DC expression of *Atg16l1* in shaping the response of the immune system to HFD-induced atherosclerosis.

### Atg16l1 Deficiency in DCs Promotes Accumulation of CD4^+^ Tregs in the Aortas of HFD fed Ldlr^−/−^ Mice and Limits the Development of Atherosclerosis

**Figure 3.**
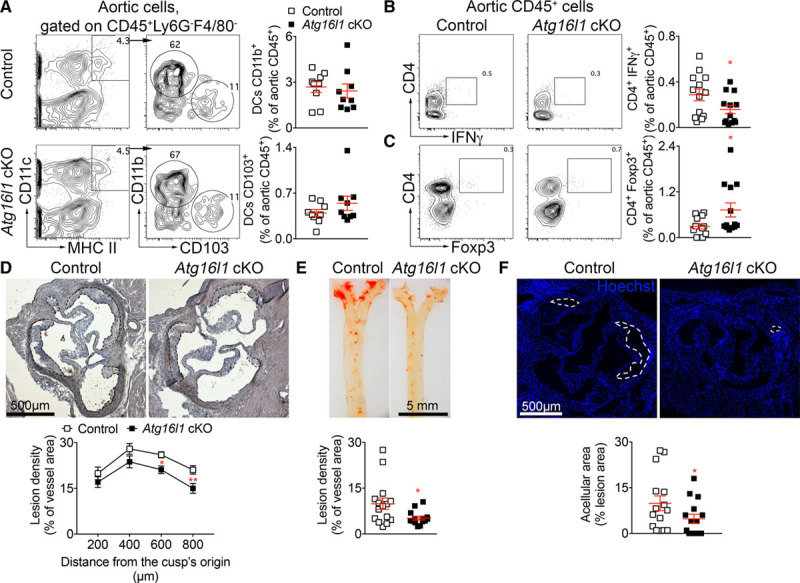
**Atg16l1 deficiency in CD11c-expressing cells promotes a shift of the effector/regulatory T cell balance in the aorta towards an antiatherogenic phenotype in Ldlr−/− (low-density lipoprotein receptor–deficient) mice. A**, Representative flow charts and quantification of dendritic cells (DC) subsets in the aorta of control and *Atg16l1* conditional knock out (cKO) mice after 8 wk of high-fat diet (HFD; n=9 mice per group). **B** and **C**, Representative flow charts and quantification of IFNγ (interferon γ)–expressing T cells (**B**) and CD4^+^ regulatory T cells (**C**) in the aortas of control and *Atg16l1* cKO mice after 8 wk of HFD (n=9 mice per group). **B**, *P=0.0441 %CD4^+^ IFNγ^+^ within aortic CD45^+^ cells from control vs *Atg16l1* cKO mice, Mann-Whitney test. **C**, *P=0.0454, %CD4^+^ Foxp3 (forkhead box P)^+^ within aortic CD45^+^ cells from control vs *Atg16l1* cKO mice, Mann-Whitney test. Graphs represent data pooled from 2 independent experiments. **D** and **E**, Representative pictures of Oil red O staining and quantification of atherosclerotic lesions in the aortic sinus (**D**) and in the en face thoracic aorta (**E**) of control (n=15 mice) and *Atg16l1* cKO (n=15 mice) mice after 8 wk of HFD. **D**, *P*=0.0002 between genotypes, 2-way ANOVA followed by uncorrected Fisher test; *P=0.0356 **P=0.0098 control vs *Atg16l1* cKO mice at indicated levels. **E**, *P=0.0411 control vs *Atg16l1* cKO mice, Mann-Whitney test. **F**, Representative pictures of Hoechst staining and quantification of acellular area in the atherosclerotic lesions of control and *Atg16l1* cKO mice after 8 wk of HFD (n=15 mice/group). *P=0.0454, control vs *Atg16l1* cKO mice, Mann-Whitney test. **D**–**F**, Data are pooled from 2 independent experiments.

Analysis of immune cell composition of aortas from *Atg16l1* cKO and control *Ldlr*^−/−^ mice showed no differences in the accumulation of DC subsets (Figure 3A), macrophages (Online Figure VIA), or T cells (Online Figure VIB). However, *Atg16l1* cKO *Ldlr*^−/−^ mice showed a significant reduction in the proportion of aortic CD4^+^ IFNγ^+^ cells, within the CD45^+^ cell population, as compared to control *Ldlr*^−/−^ mice (Figure 3B). No differences in aortic CD8^+^ IFNγ^+^ cells were observed between the 2 groups (Online Figure VIC). In parallel, we found an enrichment in CD4^+^ Tregs within aortic CD45^+^ cells of *Atg16l1* cKO compared with control *Ldlr*^−/−^ mice (Figure 3C). The predominant accumulation of CD4^+^ Tregs over T helper cells type 1 (Th1) in the aortas of *Atg16l1* cKO *Ldlr*^−/−^ mice was associated with a significant reduction of atherosclerosis development at the level of the aortic root (Figure 3D) and in the en face thoracic aorta (Figure 3E). On aortic cross-sections, we found no statistically significant differences in percentages of monocyte/macrophage-2^+^ foam cells (Online Figure VID), infiltrated CD3^+^ cells (Online Figure VIE), and αSMA (alpha smooth muscle actin)^+^ SMCs (Online Figure VIF) between the 2 groups of mice. However, acellular necrotic core area within the lesions was significantly smaller in *Atg16l1* cKO compared to control *Ldlr*^−/−^ mice (Figure 3F).

### T-Cell Depletion Abolishes the Atheroprotective Effect Associated With ATG16L1 Deficient DCs

**Figure 4.**
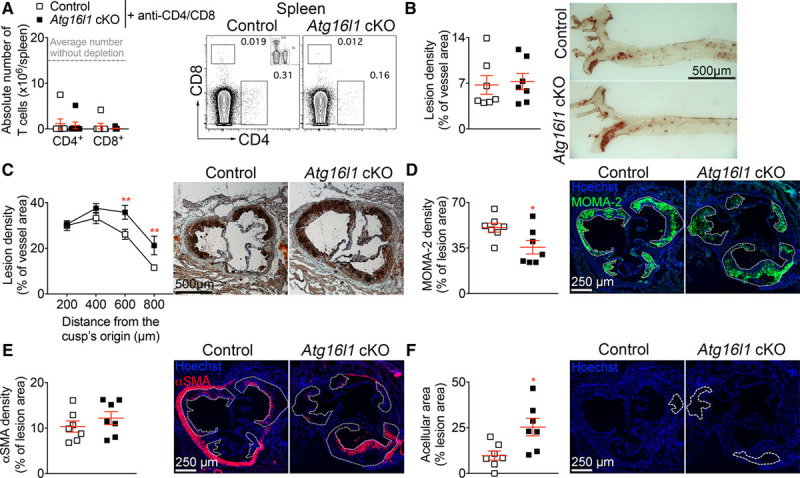
**T-cell depletion abrogates the atheroprotective effect of *Atg16l1* deficiency in *CD11c*-expressing cells. A**, Quantification and representative flow charts of CD4^+^ and CD8^+^ T cells in the spleen of control and *Atg16l1* conditional knock out (cKO) mice after 8 wk of high-fat diet (HFD) and treatment with anti–CD4/CD8 (150 µg/mouse, IP, weekly). Inset shows a representative staining of CD4 and CD8 in a nondepleted mouse. **B** and **C**, Representative pictures of Oil red O staining and quantification of atherosclerotic lesions in the en face thoracic aorta (**B**) and in the aortic sinus (**C**) of control and *Atg16l1* cKO mice after 8 wk of HFD and T-cell depletion. **C**, *P*=0.0011 between genotypes, 2-way ANOVA followed by an uncorrected Fisher test: **P=0.008 level 600 µm, **P=0.0076 level 800 µm, control vs *Atg16l1* cKO treated with anti–CD4/CD8. **D**–**F**, Quantification and representative pictures of the density (% of positive staining area to total lesion area) of monocyte/macrophage (MOMA)-2 staining (**D**, foam cells, *P=0.0408, Mann-Whitney test, control vs *Atg16l1* cKO mice), αSMA (alpha smooth muscle actin) staining (smooth muscle cells, **E**) and acellular area (**F**, *P=0.0111, Mann-Whitney test, control vs *Atg16l1* cKO mice), by immunofluorescent microscopy, on aortic sinus cross-sections from control and *Atg16l1* cKO mice after 8 wk of HFD and T-cell depletion. In **D** and **E**, small pointed line depicts the atherosclerotic lesion. In **F**, dashed line depicts the acellular area. **A**–**F**: n=7 mice/group. Data were obtained from one experiment.

The major changes in the immune response associated with atheroprotection occurred in T cells. Because a reduction in Th1 and an increase in Treg responses may confer a strong atheroprotective effect,^24,25^ we investigated the requirement for T cells in mediating atheroprotection in *Atg16l1* cKO mice. We repeated the experiment in the presence of T-cell depleting antibodies (anti-CD4/anti-CD8). No statistical difference was found in the extent of splenic T-cell depletion achieved in *Atg16l1* cKO and control *Ldlr*^−/−^ mice (Figure 4A). Treatment with anti-CD4/anti-CD8 antibodies also led to CD8α^+^, but not CD11b^+^, DC depletion in spleen (data not shown). T-cell depletion abolished the atheroprotection associated with autophagy-deficient DCs in the thoracic aorta (Figure 4B) and even led to a bigger lesion size at the level of the aortic root in *Atg16l1* cKO compared with control *Ldlr*^−/−^ mice (Figure 4C). Lesions of T-cell–depleted *Atg16l1* cKO *Ldlr*^−/−^ mice contained significantly less foam cells (Figure 4D), similar (*P*>0.05) amount of αSMA^+^ SMCs (Figure 4E) but significantly bigger acellular necrotic core (Figure 4F) compared with lesions of control *Ldlr*^−/−^ mice, consistent with their bigger size and advanced stage of development.

### Atg16l1 Deficiency in CD8α^+^ (and Related CD103^+^) DCs Does Not Impact CD4^+^ Tregs and Does Not Protect From Atherosclerosis

**Figure 5.**
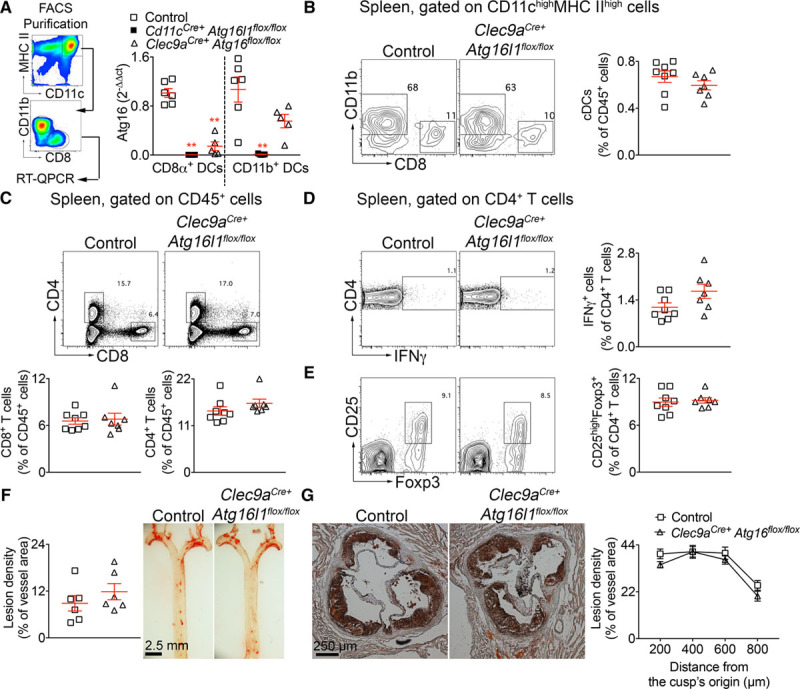
**Atg16l1 deficiency in CD8α+ dentritic cells (DCs) does not induce immune tolerance and does not protect against atherosclerosis in Ldlr^−^/^−^ (low-density lipoprotein receptor–deficient) mice. A**, Strategy for conventional DC (cDC) purification from the spleen of *CD11c*^*Cre+*^ *Atg16*^*flox/flox*^, *Clec9a*^*Cre+*^ *Atg16l1*^*flox/flox*^ and control mice (*CD11c*^*Cre−*^ *Atg16*^*flox/flox*^ and *Clec9a*^*Cre−*^ *Atg16*^*flox/flox*^) and analysis of *Atg16l1* expression by real-time quantitative polymerase chain reaction (RT-QPCR; normalized on 36B4). n=5-6 mice/group; *P*<0.0001 for comparisons between all groups (Kruska-Wallis); post hoc uncorrected Dunn’s test, ***P=0.0003 DC CD8α^+^ *CD11c*^*Cre+*^ *Atg16*^*flox/flox*^; ***P=0.0008 DC CD8α^+^ *Clec9a*^*Cre+*^ *Atg16*^*flox/flox*^ vs DC CD8α^+^ control; *P=0.056 DC CD11b^+^ *CD11c*^*Cre+*^ *Atg16*^*flox/flox*^ vs DC CD11b^+^ control. **B**, Representative flow chart showing the distribution of CD8α^+^ and CD11b^+^ DC subsets and the quantification of the proportion of cDCs in the spleens of *Clec9a*^*Cre+*^ *Atg16*^*flox/flox*^ and control mice after bone marrow transplantation (BMT) in *Ldlr*^−/−^ mice and 8 wk of high-fat diet (HFD). **C**, Representative flow chart showing the distribution of CD8^+^ and CD4^+^ T cells and the quantification of the proportion of each subset in the spleens of *Clec9a*^*Cre+*^ *Atg16*^*flox/flox*^ and control mice after BMT in *Ldlr*^−/−^ mice and 8 wk of HFD. **D**, Representative flow chart showing the proportion of IFNγ (interferon γ)-producing CD4^+^ T cells, and the quantification, in the spleens of *Clec9a*^*Cre+*^ *Atg16*^*flox/flox*^ and control mice after BMT in *Ldlr*^−/−^ mice and 8 wk of HFD. **E**, Representative flow chart showing the proportion of CD4^+^ regulatory T cells (CD3^+^CD4^+^Foxp3 [forkhead box P]^+^CD25^high^), and the quantification in the spleens of *Clec9a*^*Cre+*^ *Atg16*^*flox/flox*^ and control mice after BMT in *Ldlr*^−/−^ mice and 8 wk of HFD. **F** and **G**, Quantification and representative pictures of Oil red O staining for atherosclerotic lesions analysis on en face thoracic aorta (**F**) and in the aortic sinus (**G**) of *Clec9a*^*Cre+*^ *Atg16*^*flox/flox*^ and control mice after BMT in *Ldlr*^−/−^ mice and 8 wk of HFD. **B**–**E**, **G**, Seven to 8 mice per group. **F**, Six mice per group. Data are representative of 2 independent experiments. FACS indicates fluorescence-activated cell sorting; and MHC, major histocompatibility complex.

To gain further insights into the DC subset responsible for the atheroprotective effect, we used *Clec9a*^*Cre+*^ mice to target committed DC precursors and their progeny (cDCs but not plasmacytoid DCs nor monocyte-derived DCs.^26^ We first examined the efficiency of *Atg16l1* deletion in fluorescence-activated cell sorting (FACS)–purified splenic CD8α^+^ DCs and CD11b^+^ DCs. We found that whereas *Cd11c*^*Cre+*^ completely abrogated the expression of *Atg16l1* in both CD8α^+^ and CD11b^+^ DCs, *Clec9a*^*Cre+*^ was efficient only in CD8α^+^ DCs (≈80% reduction of *Atg16l1* expression; Figure 5A). This is consistent with the high expression of Clec9a on splenic CD8α^+^ DCs. Splenic CD11b^+^ cDCs do not express Clec9a but a previous study showed that they derive from a Clec9a-expressing committed DC precursor.^26^ Our finding that *Atg16l1* expression was only partially reduced (≈50%, *P*=0.08, control CD11b^+^ DC versus *Clec9a*^*cre+*^ *Atg16l1*^*flox*^ CD11b^+^ DC) in splenic CD11b^+^ DCs is consistent with the fact that only 50% of splenic CD11b^+^ DCs showed YFP (yellow fluorescent protein) labeling in *Clec9a*^*Cre+*^ *Rosa*^*+/EYFP*^ mice because of incomplete penetrance of Cre-mediated recombination in DC precursors.^26^ Thus, *Clec9a*^*Cre+*^ mice allow to efficiently manipulate gene expression only in CD8α^+^ (and related CD103^+^) cDCs. Bone marrow transfer experiments into irradiated *Ldlr*^−/−^ mice using *Atg16l1*^*flox/flox*^ *Clec9a*^*Cre+*^ or control bone marrow cells revealed no differences in the proportions of splenic DC subsets (Figure 5B) and CD4^+^ and CD8^+^ T cells (Figure 5C) nor in the percentages of CD4^+^ Tregs and Th1 cells (Figure 5D and 5E) after 8 weeks of HFD. This phenotype was not associated with any difference in atherosclerosis development (Figure 5F and 5G). Aortic accumulation of immune cells analyzed by flow cytometry (Online Figure VII), and plaque composition studied using immunofluorescent microscopy (foam cells, T cells, SMCs, and acellular area) were similar (*P*>0.05) between the 2 groups (Online Figure VIII). Finally, lipid profiles and weight gain did not differ between the 2 groups (Online Figure IX). Thus, the atheroprotective phenotype associated with autophagy deficiency in CD11c^+^ is not due to autophagy deficiency in CLEC9a^+^ progenitor derived cDCs (including CD8α^+^ cDCs) but is likely to be explained by a deficiency of autophagy in conventional and monocyte-derived CD11b^+^ DCs.

### Atg16l1 Deficiency Differentially Alters Gene Expression in CD8α+ and CD11b+ DCs in Ldlr^−/−^ Mice Fed an HFD

**Figure 6.**
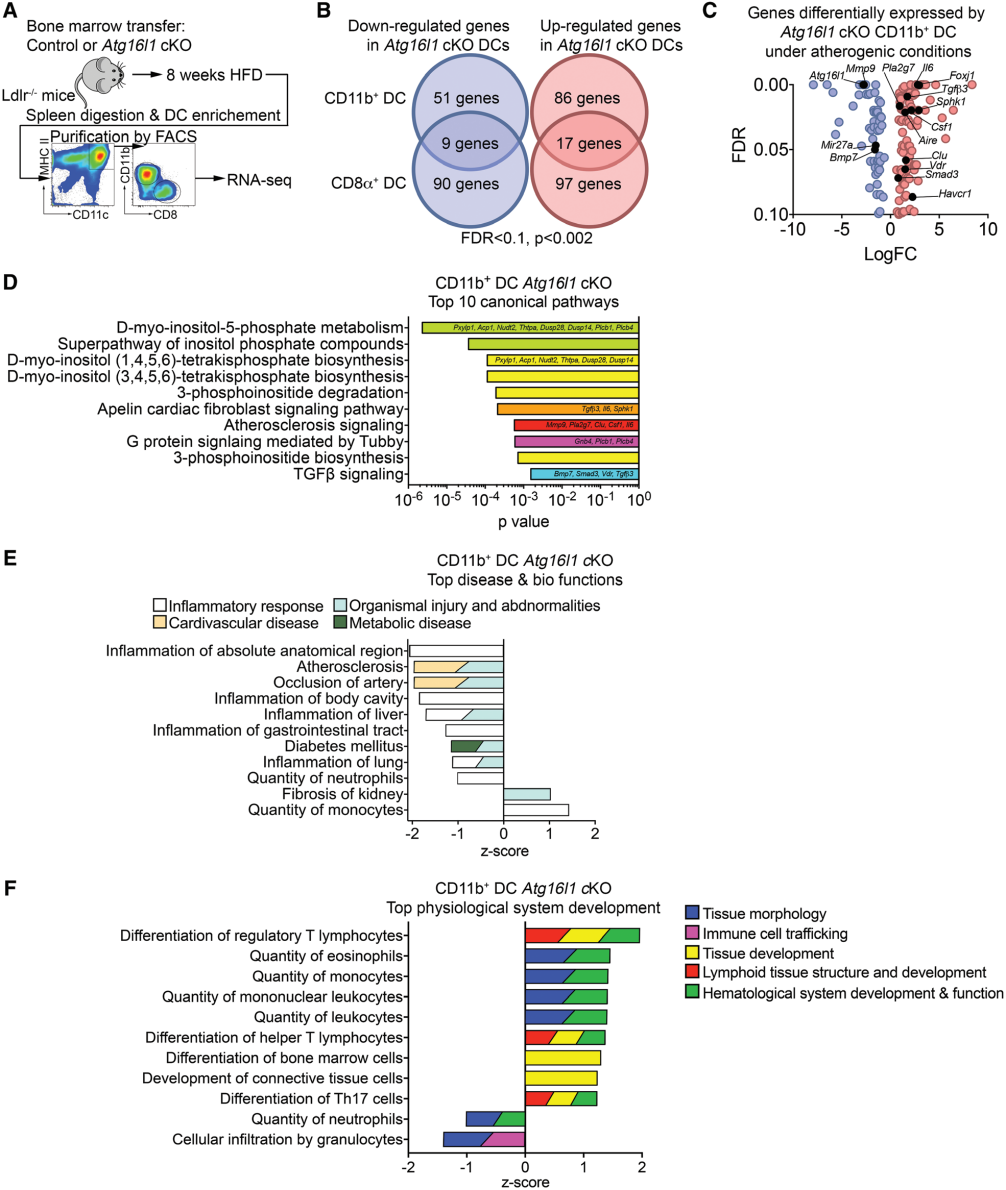
**Atg16l1 deficiency in CD11b^+^ dendritic cells (DCs) under atherogenic conditions promotes a tolerogenic gene expression program. A**, Experimental strategy for the purification of DCs before RNA sequencing (RNA-seq). DCs were purified from 3 control mice and 2 *Atg16l1* conditional knock out (cKO) mice. **B**, Venn diagram showing the proportion of differentially expressed genes between CD11b^+^ and CD8α^+^ DC, normalized on the level of expression from control DCs (false discovery rate [FDR]<0.1, *P*<0.002). **C**, Volcano plot showing the level of expression of the differentially expressed genes from CD11b^+^ DCs *Atg16l1* cKO compared with wild-type CD11b^+^ DCs (FDR<0.1, *P*<0.002). **D**, Ingenuity analysis of the top 10 canonical pathways in CD11b^+^ DCs of *Atg16l1* cKO mice. **E**, Ingenuity analysis of the top disease and bio-functions categories in CD11b^+^ DCs of *Atg16l1* cKO mice. **F**, Ingenuity analysis of the top physiological system development categories in CD11b^+^ DCs of *Atg16l1* cKO mice. FACS indicates fluorescence-activated cell sorting; FDR, ; HFD, high-fat diet; MHC, major histocompatibility complex; TGF, transforming growth factor; and Th17, T helper cells type 17.

To further understand how *Atg16l1* deficiency in CD11b^+^ but not CD8α^+^ DCs favors the expansion of CD4^+^ Tregs upon HFD feeding in *Ldlr*^−/−^ mice and prevents atherosclerosis, we purified splenic DCs (after bone marrow transplantation and 8 weeks of HFD) and analyzed their transcriptomic signature by RNA sequencing (Figure 6A and Online Table I). We found that *Atg16l1* deficient CD8α^+^ and CD11b^+^ DCs differentially expressed 215 and 165 genes, respectively, as compared with their respective wild-type (WT) control DCs (Figure 6B and 6C). However, only 10% to 15% of those genes were up or downregulated in both subsets in the absence of ATG16L1 (Figure 6C and Online Table I). Ingenuity pathway analysis further highlighted the differential impact of autophagy in CD8α^+^ versus CD11b^+^ DCs. The top canonical pathways enriched in *Atg16l1* deficient versus WT CD11b^+^ DCs (Figure 6D) corresponded to inositol (pyro)phosphate metabolism and phosphoinositide biosynthesis and degradation pathways, with major importance as cell signals in several biological processes, particularly relevant to the interface between cell signaling, membrane traffic and autophagy^27–29^ as well as immune cell functions.^30,31^ The other major canonical pathways relate to atherosclerosis signaling and TGF (transforming growth factor)-β signaling of high relevance to the immune response in atherosclerosis. Accordingly, the most enriched disease and biological functions categories corresponded to inflammatory response, cardiovascular disease (atherosclerosis and occlusion of artery), and metabolic disease (diabetes mellitus), with a negative *Z* score indicating decreased bio-function in the absence of ATG16L1, and the most enriched physiological system development and function categories corresponded to lymphoid tissue structure and development, with a positive *Z* score for differentiation of Tregs, indicative of increased bio-function. None of these pathways was differentially expressed in *Atg16l1* deficient versus WT CD8α^+^ DCs (Online Figure X). Further analysis revealed that *Atg16l1* deficient CD11b^+^ DCs upregulated the expression of several genes implicated in immune tolerance and CD4^+^ Treg expansion and function (Figure 6E), such as *Vdr*, *Aire*, *Foxj1*, *Sphk1*, *Havcr1* (also known as *Tim1*), *mir27*, *Tgfb3* and *Smad3*, most of which did not show significant differential expression in *Atg16l1* deficient versus WT CD8α^+^ DCs (Online Table I). Thus, autophagy has a selective impact on CD11b^+^ DCs with a major implication for their tolerogenic potential under HFD feeding in *Ldlr*^−/−^ mice.

### Splenic CD11b+ DCs Promote Antigen-Specific CD4+ Treg Expansion in the Presence of TGF-β

**Figure 7.**
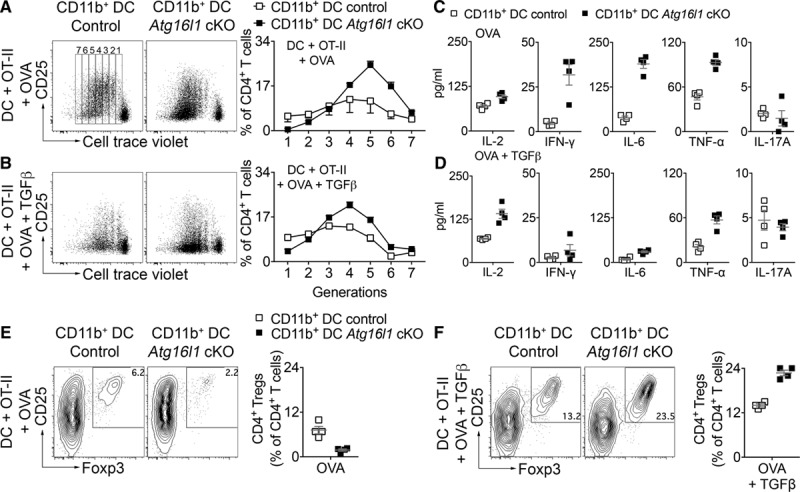
**Atg16l1 deficiency in CD11b^+^ dendritic cells (DCs) promotes polarization of antigen-specific CD4^+^ regulatory T cell (Treg) under TGF (transforming growth factor) β supplementation in vitro**. **A** and **B**, Representative flow chart and quantification of naive OTII cell proliferation (using cell trace violet, generations are shown in **A**) after coculture with OVA (ovalbumin) protein and fluorescence-activated cell sorting-purified (control and *Atg16l1* conditional knock out [cKO]) CD11b^+^ DC in the absence (**A**) or presence of TGFβ (**B**). Data were obtained using technical replicates and are representative of 2 independent experiments. **C** and **D**, Cytokine quantification by multiplex cytometric bead array beads of IL (interleukin) 2, IFN (interferon) γ, TNF (tumor necrosis factor), IL6, and IL17 in the supernatants of naive CD4+ OTII T cells cocultured for 5 d with CD11b^+^ DCs from control or *Atg16l1* cKO mice in the presence of OVA±TGFβ. Data were obtained using technical replicates and are representative of 2 independent experiments. **E** and **F**, Representative flow chart and quantification of the proportion of CD4^+^ Tregs (Foxp3 [forkhead box P]^+^CD25^high^) generated from naive OTII CD4^+^ T cells after 5 days of coculture with control or *Atg16l1* cKO CD11b^+^ DC with OVA only (**E**) or OVA+TGFβ(**F**). Data were obtained using technical replicates and are representative of 2 independent experiments.

To examine the ability of *Atg16l1* deficient and WT splenic CD11b^+^ DCs to induce CD4^+^ Tregs in vitro, we first used an antigen-independent approach. Naive (CD62L^high^, CD44^neg^, CD25^neg^) CD4^+^ T cells (from C57Bl/6J mice) were purified by flow cytometry (99% purity) and cultured with FACS-purified CD11b^+^ DCs (99% purity) and soluble anti-CD3±TGF-β. In these conditions, we found no differences between WT and *Atg16l1* deficient DCs in the induction of CD4^+^ Tregs (Online Figure XI). We then used antigen-specific naive OTII CD4^+^ T cells, which express a TCR specific to chicken OVA (ovalbumin) and cultured them with splenic CD8α^+^ or CD11b^+^ DCs in the presence of OVA protein±TGF-β. First, we found that *Atg16l1* deficient CD8α^+^ DCs were not able to induce T-cell proliferation, as compared to WT CD8α^+^ DCs (Online Figure XIIA and XIIB), in accordance with the role of autophagy in antigen presentation.^17^ Interestingly, however, *Atg16l1* deficiency did not alter antigen presentation in CD11b^+^ DCs, which induced more T-cell proliferation as compared to WT CD11b^+^ DCs (Figure 7A). The addition of TGF-β reduced the number of cell divisions particularly in the presence of *Atg16l1* deficient CD11b^+^ DCs, suggesting decreased T-cell proliferation (Figure 7A and 7B). Interestingly, OVA-treated *Atg16l1* deficient CD11b^+^ DCs seemed to enhance naive OTII cells production of IL2, IFN-γ, and IL6 in comparison with control DCs (Figure 7C). However, when TGF-β was added in the culture, OVA-treated *Atg16l1* deficient CD11b^+^ DCs were no longer able to stimulate IFN-γ production by OTII cells, promoted only a modest increase of IL6, but were still able to induce a marked production of IL2 in comparison with control DCs (Figure 7C). Coculture of naive OTII cells with *Atg16l1* deficient CD11b^+^ DCs in the absence of TGFβ seemed to prevent T-cell polarization into CD4^+^CD25^+^Foxp3 (forkhead box P3)^+^ Tregs (Figure 7D). However, the addition of TGFβ to the coculture substantially increased the polarization of naive OTII cells into CD4^+^CD25^+^Foxp3^+^ Treg cells in presence of *Atg16l1* deficient CD11b^+^ DCs compared with WT CD11b^+^ DCs (Figure 7E). This tolerogenic effect was restricted to *Atg16l1* deficient CD11b^+^ DCs in the presence of TGFβ and was not induced by incubation with *Atg16l1* deficient CD8α^+^ DCs (Online Figure XIIC and XIID). Finally, we examined the impact of *Atg16l1* deficiency on the uptake of oxLDL (oxidized low-density lipoprotein). Although the latter was not affected by *Atg16l1* deficiency in macrophages, we found a slight reduction of oxLDL uptake by *Atg16l1* deficient compared to WT DCs (Online Figure XIIE). However, the extent of oxLDL uptake was consistent between CD8α^+^ and CD11b^+^ DCs (Online Figure XIIE).

### Treatment With an Anti-CD25 Antibody Prevents HFD-Induced CD4+ Treg Expansion and Abrogates the Atheroprotective Effect of Autophagy Deficiency in DCs

**Figure 8.**
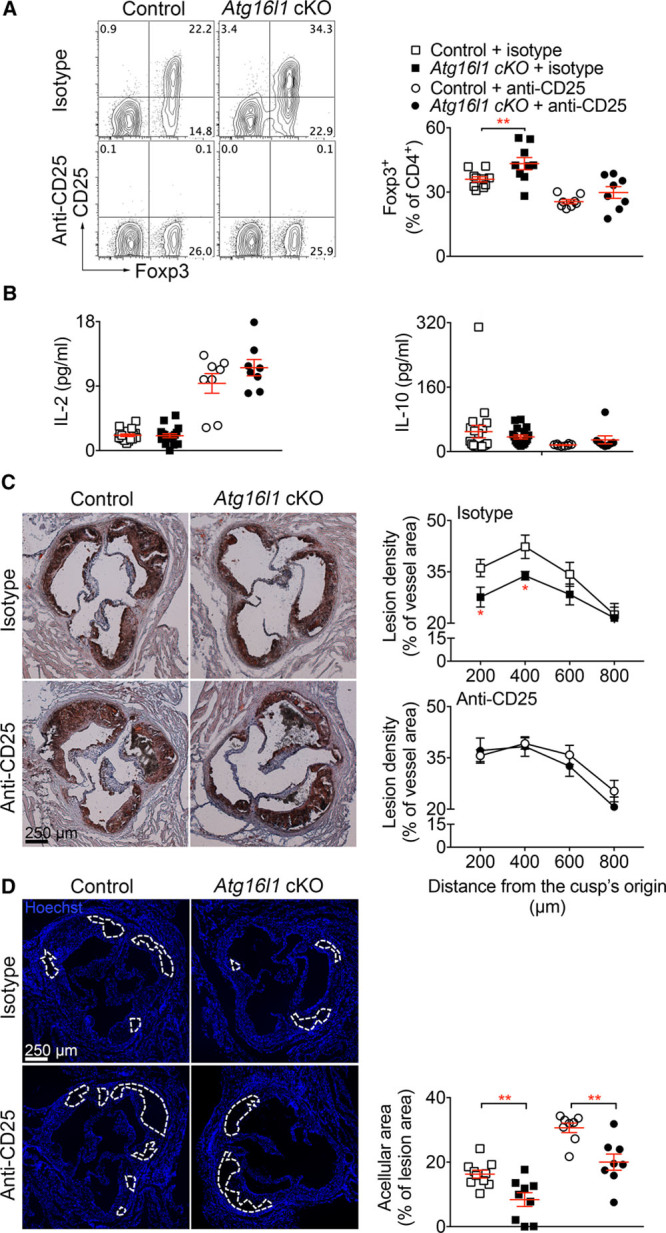
**Treatment with anti-CD25 (PC61) antibody abrogates the atheroprotective effect of Atg16l1 deficiency in CD11c expressing cells. A**, Representative flow chart and quantification of Foxp3 (forkhead box P) expressing CD4^+^ T cells in the spleens of *Atg16l1* conditional knock out (cKO) and control mice treated with isotype-matched or anti-CD25 antibody, starting at the beginning of the high-fat diet (HFD; 250µg of antibody/mouse/wk, for 8 wk). **P=0.0066, control+isotype vs *Atg16l1* cKO+isotype Mann-Whitney test; *P*=0.1393, control+anti-CD25 vs *Atg16l1* cKO+anti-CD25, Mann-Whitney test. **B**, IL (interleukin) 2 and IL10 titration in the plasma of control and *Atg16l1* cKO mice infused with isotype-matched or anti-CD25 antibody. Control+isotype, n=19; *Atg16l1 cKO*+isotype, n=18; control+anti-CD25, n=8; *Atg16l1 cKO*+anti-CD25, n=8. **C**, Representative pictures and quantification of atherosclerotic lesions using Oil red O staining at the level of the aortic sinus from *Atg16l1* cKO and control mice after bone marrow transplantation (BMT) in *Ldlr*^−/−^ (low-density lipoprotein receptor–deficient) mice and 8 wk of HFD, and treatment with isotype-matched or anti-CD25 antibody. 2-way ANOVA followed by an uncorrected Fisher test: *P=0.0376 level 200 µm, *P*=0.0498 level 400 µm, control vs *Atg16l1* cKO treated with isotype; control vs *Atg16l1* cKO treated with anti-CD25, 2-way ANOVA *P*=0.3544. Control+isotype, n=10; *Atg16l1 cKO*+isotype, n=8; control+anti-CD25, n=8; *Atg16l1 cKO*+anti-CD25, n=8. **D**, Representative pictures and quantification of acellular area in atherosclerotic lesions of *Atg16l1* cKO and control mice after BMT in *Ldlr*^−/−^ mice and 8 wk of HFD, and treatment with isotype-matched or anti-CD25 antibody. **P=0.0041 control vs *Atg16l1* cKO mice treated with isotype, Mann-Whitney test; **P=0.003 control vs *Atg16l1* cKO mice treated with anti-CD25, Mann-Whitney test.

To examine if the atheroprotective effect is dependent on CD4^+^ Tregs, we generated *Atg16l1* cKO and control *Ldlr*^−/−^ mice and treated them with a previously validated anti-CD25 antibody (ref) or an isotype control antibody during the 8 weeks of HFD feeding. Anti-CD25 treatment significantly reduced the percentages of Tregs, which were no longer different between *Atg16l1* cKO and control *Ldlr*^−/−^ mice (Figure 8A) Systemic IL2 levels were significantly increased in mice treated with anti-CD25, further supporting the efficiency of blocking the IL2 receptor, and this was associated with a decrease of IL10 (Figure 8B), a Treg related immunosuppressive and antiatherogenic cytokine.^32,33^ We observed no differences in plasma lipid profiles or weight gain between the 4 groups of mice (Online Figure XIII). Analysis of atherosclerosis on aortic root cross-sections showed that treatment with anti-CD25 completely abrogated the atheroprotective effect associated with *Atg16l1* deficiency in DCs (Figure 8C). The analysis of plaque phenotype revealed no significant differences in foam cell and SMC contents between the groups (Online Figure XIVA and XIB). However, treatment with anti-CD25 significantly increased T-cell accumulation in *Atg16l1* cKO mice as compared to control *Ldlr*^−/−^ mice (Online Figure XIVC). In addition, anti-CD25 treatment increased the acellular area in lesions of *Atg16l1 cKO* mice (Figure 8D), suggesting a more inflammatory and complex phenotype.

## Discussion

We show here that *Atg16l1* deletion in bone marrow–derived DCs promotes Treg expansion and limits atherogenesis in *Ldlr*^−/−^ mice fed an HFD. These effects are not due to *Atg16l1* deletion in CD8α^+^ DCs but to its deletion in the other CD11c-expressing cells. The latter include CD11b^+^ DCs, plasmacytoid DCs, and some subsets of macrophages. However, ATG16L1 deletion in macrophages is unlikely to account for the atheroprotective phenotype given the previously reported proatherogenic effect of abrogation of autophagy in macrophages.^8,9^ Importantly, our data indicate that CD11b^+^ DCs acquire tolerogenic properties after *Atg16l1* deletion, which promote Treg expansion, and reduce effector T-cell accumulation and production of Th1-related cytokines in atherosclerotic lesions. Thus, in contrast to the proinflammatory and proatherogenic role of autophagy deficiency in macrophages,^7–9^ autophagy deficiency in DCs promotes a counter-regulatory immunosuppressive response that maintains vascular homeostasis in *Ldlr*^−/−^ mice under HFD and limits the development of atherosclerosis.

Previous studies established a role for autophagy in antigen processing, loading on MHCII molecules, and antigen presentation (reviewed in Ghislat and Lawrence^17^). We found that this is the case with *Atg16l1* deficient CD8α^+^ DCs, which were unable to (process and) induce antigen-specific proliferation of T cells in comparison to their WT CD8α^+^ DCs. Intriguingly, however, the ability of CD11b^+^ DCs to induce antigen-specific T-cell proliferation was not affected by *Atg16l1* deletion. In fact, we rather found increased antigen-specific T-cell proliferation in presence of *Atg16l1* deficient compared with WT CD11b^+^ DCs. Interestingly, available data in the literature indicate that knockdown of *Atg16l1* in monocyte-derived DCs may induce more T-cell proliferation in an alloreactive model,^34^ a finding attributed to increased DC numbers and expression of costimulatory molecules, and reduced expression of A20 (TNFAIP3: tumor necrosis factor, alpha-induced protein 3). However, *Atg16l1* deletion in our model did not affect DC numbers, and none of those genes was significantly altered by *Atg16l1* deletion in DCs within the context of HFD-induced atherosclerosis. Thus, the reason for the differential impact of *Atg16l1* deletion on CD8α^+^ DC-dependent versus CD11b^+^ DC-dependent antigen-specific T-cell proliferation remains unknown and merits further investigation.

Reduced T-cell priming by *Atg16l1* deficient CD8α^+^ DCs in *Ldlr*^−/−^ mice fed an HFD could have contributed to the observed decrease of aortic Th1 cells and the reduction of atherosclerosis, given the well-validated proatherogenic role of Th1 immunity.^24^ However, selective deletion of *Atg16l1* in CD8α^+^ DCs did not reproduce the phenotype. Moreover, the atheroprotection seen in *Ldlr*^−/−^ mice with *Atg16l1* deficient DCs was dependent on Tregs, which were unaltered in *Ldlr*^−/−^ mice with *Atg16l1* deficient CD8α^+^ DCs. This is consistent with the observation that CD8α^+^ DCs were unable to promote Treg expansion in vitro.

In contrast to CD8α^+^ DCs, *Atg16l1* deficiency in CD11b^+^ DCs substantially enhanced Treg generation in vitro, in the presence of TGF-β. This is a new finding, which could not be anticipated from previous observations. Xiong et al^35^ reported increased Tregs in cardiac allograft recipient mice treated with FLT3 (fms-related tyrosine kinase 3) and rapamycin compared with single treatments and suggested a role for autophagy in Treg induction. However, DCs were not the only cells targeted by rapamycin in that experiment, and rapamycin could have exerted immune effects independent of its role in autophagy regulation. Chu et al^36^ reported reduced in vitro generation of IL10^+^Foxp3^+^ Tregs in the presence of *Atg16l1* deficient versus WT bone marrow monocyte-derived DCs when the cells were incubated with outer membrane vesicles harvested from *Bacteroides fragilis*. The total number of Foxp3^+^ cells was not altered, and *Atg16l1* deficiency in DCs did not affect the generation of IL10^+^Foxp3^+^ Tregs in the absence of outer membrane vesicle. Moreover, the system does not involve antigen-specific presentation of outer membrane vesicle to T cells, but the phenotype is attributed to alteration of DC inflammatory response by outer membrane vesicle. Thus, the data of Chu et al^36^ apply to a very particular system and do not contradict nor invalidate our findings. In fact, our data initially suggested that, in the absence of TGF-β, the limited antigen-specific Treg induction that occurs in culture was rather reduced in the presence of *Atg16l1* deficient versus WT CD11b^+^ DCs. This was not in line with our RNA-sequencing data, which showed significant differential upregulation of genes and pathways involved in the regulation of immune cell activation, the generation of Tregs, and the induction of immune tolerance. A clue to this apparent discrepancy came from a closer look at the RNA-sequencing data, which revealed TGF-β signaling to be one of the most differentially upregulated pathways in *Atg16l1* deficient DCs in vivo. The high relevance of TGF-β is further attested by the importance of this cytokine in the in vivo generation and maintenance of Tregs, and the requirement for this cytokine to convert naive CD4^+^CD25^−^ T cells into Foxp3^+^ Tregs in vitro.^37^ We, therefore, repeated the in vitro Treg generation experiment in the presence of TGF-β and observed a substantial impact of *Atg16l1* deficiency in CD11b^+^ DCs in boosting antigen-specific Treg generation compared with WT CD11b^+^ DCs. Interestingly, this was associated with reduced production of IFN-γ but increased production of IL2, a cytokine that is essential for TGF-β–mediated conversion of naive CD4^+^CD25^−^ T cells into Foxp3^+^ Tregs and Treg expansion.^38^ It remains unknown why Treg generation was not enhanced by *Atg16l1* deficient DCs when polyclonal CD4^+^ T cells were used. However, the promotion of Treg expansion observed using the antigen-specific OTII system nicely supports the in vivo RNA-sequencing data and the impact of *Atg16l1* deficiency in DCs on Treg accumulation in *Ldlr*^−/−^ mice fed an HFD. Whether additional mechanisms (eg, modulation of cytokine production by DCs) contribute to the expansion of Tregs by autophagy-deficient DCs is currently unknown and will require further investigations.

A recent report found that Tregs ameliorate autoimmunity by restraining autophagy in DCs.^39^ These findings are in line with our data and further support an important impact of autophagy on the tolerogenic potential of DCs. Interestingly, *Atg16l1* was the most significantly downregulated gene after interaction with Tregs, and this occurred through CTLA-4 (cytotoxic T-lymphocyte-associated protein 4)–dependent activation of the PI3K (phosphoinositide 3-kinase)/Akt (PKB: protein kinase B)/mTOR (mammalian target of rapamycin) axis, further highlighting, as in our RNA-sequencing data, the close interconnection between inositol phosphate and 3-phosphoinositide pathways and the autophagy process, and their important consequences on DC biology and immune homeostasis.

Finally, we would like to mention that one of the most significantly downregulated genes in *Atg16l1* deficient versus WT DCs (whether CD8α^+^ or CD11b^+^) was *Slc15a2*. This gene encodes a proton-coupled oligopeptide transporter recently found to mediate the transport of bacterially derived di-and tri-peptides that activate NOD receptors in bone marrow–derived macrophages.^40^ Although the relevance to atherosclerosis remains unknown, this finding may have important implications for the mechanisms of Crohn disease. *NOD2* and *ATG16L1* genetic variants that lead to reduced function are associated with Crohn disease. Reduced NOD2 activation has been shown to inhibit autophagy in DCs altering both bacterial trafficking and MHCII-dependent antigen presentation.^41^ Our preliminary data may suggest that, in turn, reduced ATG16L1 function may inhibit bacterial dependent activation of NOD2, thereby altering the susceptibility to colitis. In this case, Treg maintenance in the absence of functional ATG16L1 may be acting as a safety break on intestinal inflammation. This hypothesis merits experimental testing.

In conclusion, we show that *Atg16l1* deficiency in murine CD11b^+^ DCs profoundly impacts their phenotype towards a tolerogenic potential in *Ldlr*^−/−^ mice fed a HFD and reduces atherosclerosis through the expansion and maintenance of atheroprotective Tregs. Our results may have implications for immune-modulatory strategies to limit atherosclerosis through selective modulation of autophagy in DCs.

## Acknowledgments

We thank Caetano Reis e Sousa, Francis Crick Institute, London, United Kingdom, for providing the *Clec9a*^*+/cre*^ mice.

## Sources of Funding

This study was supported by the British Heart Foundation (CH/10/001/27642 and Grant No. 1659), the European HEALTH 2013.1.3-3 programme, HORIZON2020/ERC Grant agreement no. 648889 (A.K.), and the Wellcome Trust (senior investigator award 106260/Z/14/Z to A. Kaser).

## Disclosures

None.

## Supplementary Material

## Novelty and Significance

### What Is Known?

- Autophagy in macrophages limits atherosclerosis.
- Dendritic cells (DCs) modulate the development of atherosclerosis. However, the role of autophagy in DC function during atherosclerosis is unknown.
