# Supplementary material for: Impaired Autophagy in CD11b+ Dendritic Cells Expands CD4+ Regulatory T Cells and Limits Atherosclerosis in Mice
Source: Circ Res. 2019 Nov 7;125(11):1019–34. doi: 10.1161/CIRCRESAHA.119.315248 (PMC6844650; doi:10.1161/CIRCRESAHA.119.315248)
Supplement: Supplementary file 3 [file res-125-1019-s003.pdf]

SUPPLEMENTAL MATERIAL

Clement M et al.,

Impaired autophagy in CD11b$^+$ dendritic cells expands CD4$^+$ regulatory T cells and limits atherosclerosis in mice

Expanded Methods

Animals

All the experiments were approved by the local ethics committee and were performed under Home Office, UK license PA4BDF775. All the mice were on a C57Bl/6J genetic background. *Ldlr*$^{-/-}$*, LC3-GFP, Atg16l1*$^{flox/flox}$*, Cd11c-cre, Clec9a-cre* mice were already described. We used female *Ldlr*$^{-/-}$ mice (6-8 week-old) for our studies given that previous studies on the role of macrophage autophagy in atherosclerosis were performed using female *Ldlr*$^{-/-}$ mice [1]. The mice were lethally irradiated (9.5 Gy), then injected i.v. (tail vein) with 1x10^7 bone marrow cells from donor mice. After 4 weeks of recovery, mice were fed a chow or high fat diet (21 % Fat, 0.15 % Cholesterol, Special Diet Services) for 8 weeks. Female littermate *CD11c*$^{Cre-}$ *Atg16l1*$^{flox/flox}$ and *CD11c*$^{Cre+}$ *Atg16l1*$^{flox/flox}$, as well as female littermate *Clec9a*$^{Cre-}$ *Atg16l*$^{flox/flox}$ and *Clec9a*$^{Cre+}$ *Atg16l1*$^{flox/flox}$, were used as bone marrow donors to reconstitute lethally irradiated *Ldlr*$^{-/-}$ animals. Chloroquine injections (i.p., 10mg/Kg, as described in [2]) were performed during the 8th week of HFD, 48 and 24 hours before the analysis. Chloroquine is not specific for autophagy but it raises the lysosomal pH and interrupts the autophagosome-lysosome fusion step, and is a well-validated agent for acute modulation and interrogation of the autophagic flux [3].

T cell depletion was performed using anti-CD4 (clone: YTS 191) and anti-CD8 (clone: YTS 169.4) antibodies, i.p. injection of 150 µg of each antibody/mouse once a week (Bioxcell), starting the day before the HFD. Injections of anti-CD25 (clone: PC-61, Bioxcell), as well as isotype (clone: HRPN, Bioxcell), were started the day before the high fat diet, 250 µg of antibody/mouse, once a week, for 8 weeks.

Flow cytometry

Spleen cell suspension were obtained after meshing and red blood cell lysis and were incubated with Fc block solution (ebiosciences, clone 93, dilution 1/200 in flow buffer containing PBS, 1 % BSA, 2 mM EDTA, 0.01 % NaN3) for 10 minutes at 4°C. Cells were then stained with fluorescently labelled anti-mouse antibodies, diluted in flow buffer at indicated concentrations (**Online Table I**) for 30 minutes at 4°C. Cells were extensively washed in PBS and analyzed by flow cytometry. Intracellular cytokine staining was performed after 6 hours of stimulation of the cell suspensions with leukocyte activation cocktail (BD Biosciences) in complete medium. Cells were extensively washed, fixed and permeabilized with solutions from the Fix/Perm buffer kit (BD Biosciences). Cells were then stained as described previously [4]. For the analysis of $CD4^+$ Tregs, surface staining was performed before fixation, permeabilization and intracellular staining of Foxp3, using the Foxp3 staining kit (Thermofisher).

For aortic cell analysis, aortas were extensively perfused to remove any trace of blood. Adventitia was carefully removed from the media under a dissecting microscope, on ice. Aortas were then minced, and thoroughly mixed with a digestion solution (Collagenase D 0.2 mg/ml, Dispase I 0.2 U/ml, Elastase 1 mg/ml [Worthingthon], DNAse I 0.2 mg/ml diluted in RPMI 1640). Samples were incubated at 37°C, under agitation, for 40 minutes. Cell suspensions were pipetted up and down until disappearance of all residual piece of tissue and filtered through 70 µm cell strainer. After extensive washes in PBS, cells were stained for flow cytometry or stimulated in vitro for intracellular cytokine detection.

Flow cytometric acquisition was performed on a LSR II Fortessa (BD biosciences) equipped with 4 lasers (405, 488, 561 and 640 nm). Cell analysis was done using BD

FACSDiva Software 6.0 and figure-displayed dot plots and histograms were obtained using FlowJo software (TreeStar). CBA were analyzed using FCAP array v3.0.

Immunofluorescence

Cryosections (of PFA fixed aortas, conserved at -80°C) were dried for 30 minutes at room temperature before being rehydrated in PBS for 10 minutes before the staining. Sections were then permeabilized in 0.1 % Triton X-100, 0.1 % Citrate buffer pH 6.0 (Dako) for 30 minutes. They were then washed in PBS, and incubated with the blocking solution (flow buffer + 5 % serum of secondary antibody species, i.e. goat or donkey) for 30 minutes, before being incubated with primary antibodies diluted in the blocking solution at 5 µg/ml **(Online table III)** overnight at 4°C. Samples were extensively washed with PBS and incubated with secondary antibodies diluted in the blocking solution at 1/200-1/400 **(Online table III)** for 4 hours. Samples were washed again in PBS, nuclei were stained with Hoechst 33342 (Invitrogen) and samples were mounted with CC mount^{TM} (Sigma). Confocal analysis of samples was done using a Carl Zeiss LSM 700 confocal microscope and Zen2009 software. Epifluorescence analysis and brightfield imaging were done using Leica DM6000B microscope and analyzed with accompanying software. Exposure times were set using control sections stained with the secondary antibodies in the absence of the primary antibodies. Images analysis was performed using Adobe Photoshop CS5 and ImageJ (NIH).

Plaque quantification

For atherosclerotic plaque analysis, tissues were fixed in PFA 4% on the day of the sacrifice, and then samples were kept in PBS at 4°C. Oil red O staining and quantifications were performed in a blinded manner as previously described [5].

Real time quantitative PCR

DCs were FACS-purified in RLT buffer ($2x10^5$ cells in 300µl). RNA purification was performed using RNeasy mini kit (Qiagen). Isolated RNA (≥100 ng) was reverse-transcribed using the QuantiTect Reverse Transcription Kit (Qiagen). Real Time PCR was performed using 5 µl of cDNA product, (diluted 5 times) using MESA Green qPCR master mix (Eurogentec), on a Roche Lightcycler. Primers used: 36B4: for 5’->3’ TCTCCAGAGGCACCATTGAAA; 36B4: rev 5’->3’ CTCGCTGGCTCCCACCTT-5’; *Atg16l1* for 5’->3’: AGGCGTTCGAGGAGATCATT; *Atg16l1* rev 5’->3’: CATTCCACGCACCATCATGT.

RNA sequencing

Library preparation and RNA sequencing. RNA extraction and residual DNA removal upon DNase digestion was performed with the RNeasy mini kit (Qiagen) according to the manufacturer’s instructions. 7 ng of total RNA, after quality check with Agilent Bioanalyser 2100 system, was made into sequencing libraries using Takara’s the SMARTer Stranded Total RNA-Seq Kit v2 – Pico Input Mammalian, following manufacturer’s instruction, at the Genomics / Transcriptomics Core (IMS-MRL, Cambridge, UK). Briefly, total RNA was fragmented before reverse transcription. Second strand cDNA was PCR synthesized with the incorporation of SMART technology. cDNA originally from rRNA was removed selectively before Illumina-compatible barcoded libraries were generated via PCR amplification. Indexed libraries were normalized, pooled and were sequenced on the Illumina HiSeq 4000 platform, single-end reads (SE50) at the Genomics Core Facility, Cancer Research UK Cambridge Institute (Cambridge, UK).

Sequencing Bioinformatics and Differential Gene Expression. Sequence reads were mapped to the mouse genome (GRCm38) and gene-level count was performed using Star 2.5.0a using standard parameters. The counts were then imported into EdgeR, a GLM model was used to determine differential expressed genes (DEGs) amongst different conditions.

Top canonical pathways, diseases and bio-functions. The analysis of DEGs with an FDR<0.1 was performed with the Core analysis module from the Ingenuity Pathways Analysis system (IPA, http://www.ingenuity.com). The top 10 canonical pathways were selected for each data set. In addition, the Core analysis module of the IPA software allows to identify top diseases and biofunctions related to DEGs. In our data set, most of the DEGs in CD11b+ DCs from Atg16l1 cKO were implicated in diseases and disorders, and physiological system development and functions related to inflammation and atherosclerosis. From the sections "diseases and disorders" and "physiological system development" we chose to depict bio-functions/pathways with an -1>z-score>1.

T cell proliferation

Primary mouse naïve $CD4^+$ T cells (from WT and OT-II mice) and DCs (purification after digestion with Collagenase D 0.2 mg/ml (Sigma), DNAse I 0.2 mg/ml (Sigma), Dispase I 0.2 U/ml (stemcell), from littermate $Cd11c^{Cre+}$ $Atg16^{flox/flox}$ and $Cd11c^{Cre-}$ $Atg16^{flox/flox}$) were enriched from spleen cell suspensions using Milteny kits and AutoMACS. Cells were then stained with a cocktail of antibodies (naïve $CD4^+$ T cells: anti-CD4, anti-CD62L, anti-CD44; DCs: anti-CD11c, anti-MHC II, anti-CD11b, anti-CD8α) and purified by flow cytometry. Cells were cultured at 37°C and in 5 % CO2 in a humidified incubator in complete medium (RPMI 1640 containing L-glutamine + 10 % [vol/vol] heat-inactivated FBS, 100 IU/ml penicillin, 100 µg/ml streptomycin and 0.05 mM β-mercaptoethanol) at a ratio of 1/5 (DC/T cells, with a minimum of $5x10^3$ DC/well). Anti-CD3 stimulation was achieved using 10 µg/ml of antibody for 5 days (clone 145-2C11, Biolegend). OVA protein (Sigma) was used at a final concentration of 20 µg/ml. TGFβ1 (Biolegend) was used at a final concentration of 10 ng/ml.
On day 5, plates were centrifuged and supernatants were frozen for further analysis, whereas cells were analyzed by flow cytometry. Cell proliferation was analysed using Cell trace violet (Thermofisher) and staining was performed according to the manufacturer's instructions. Cell Trace violet dilution and generations were analyzed with FlowJo, using the proliferation module. Cytokines were quantified using mouse Th1/Th2/Th17 CBA kit (BD Biosciences) according to the manufacturer's instructions.

Plasma titration

Blood and plasma collection, as well as lipid profile analysis were performed as described previously [6]. Cytokines were titrated using Mouse U-plex for IL-2 and IL-10 from MESO SCALE Discovery. Isotyping was performed using the mouse isotyping Panel 1 from MESO SCALE Discovery. Chemiluminescent ELISA was used as previously described to determine antibody titers to specific antigens in plasma [7, 8].

Statistics

Statistical analyses were performed using the Graphpad Prism 7 software. Non-parametric Mann-Whitney test was performed to compare two groups; non-parametric Kruskal-Wallis test was performed, followed by uncorrected Dunn's test, to compare

more than 2 groups. For analysis of atherosclerotic lesions in the aortic sinus, we have assumed a normal distribution of the data based on a large published dataset in this animal model, and have estimated that n=7 mice per group would be required to detect 30% difference in lesion size (mean=120,000, SD=22,000) with 0.80 power. Two-way ANOVA was performed, followed by uncorrected Fisher's test. Data were expressed as Mean ± SEM, differences were considered significantly different $p<0.05$. All main outcome data are presented in figures as individual data plots. Thus, numeric data are not provided in addition to the figures. However, they are available upon request.

Data availability

The data for the findings of this study are available from the corresponding author upon reasonable request. RNA–seq data are available at the Gene Expression Omnibus (GEO) under accession number GSE137760.

References

1. Liao X, Sluimer JC, Wang Y, Subramanian M, Brown K, Pattison JS, Robbins J, Martinez J, Tabas I. Macrophage autophagy plays a protective role in advanced atherosclerosis. *Cell Metab*. 2012;15:545-553.
2. Ravindran R, Loebbermann J, Nakaya HI, et al. The amino acid sensor GCN2 controls gut inflammation by inhibiting inflammasome activation. *Nature*. 2016;531:523-527.
3. Klionsky DJ, Abdelmohsen K, Abe A, et al. Guidelines for the use and interpretation of assays for monitoring autophagy (3rd edition). *Autophagy*. 2016;12:1-222.
4. Clement M, Haddad Y, Raffort J, Lareyre F, Newland SA, Master L, Harrison J, Ozsvar-Kozma M, Bruneval P, Binder CJ, Taleb S, Mallat Z. Deletion of IRF8 (Interferon Regulatory Factor 8)-Dependent Dendritic Cells Abrogates Proatherogenic Adaptive Immunity. *Circ Res*. 2018;122:813-820.
5. Taleb S, Romain M, Ramkhelawon B, Uyttenhove C, Pasterkamp G, Herbin O, Esposito B, Perez N, Yasukawa H, Van Snick J, Yoshimura A, Tedgui A, Mallat Z. Loss of SOCS3 expression in T cells reveals a regulatory role for interleukin-17 in atherosclerosis. *J Exp Med*. 2009;206:2067-2077.
6. Clement M, Basatemur G, Masters L, Baker L, Bruneval P, Iwawaki T, Kneilling M, Yamasaki S, Goodall J, Mallat Z. Necrotic Cell Sensor Clec4e Promotes a Proatherogenic Macrophage Phenotype Through Activation of the Unfolded Protein Response. *Circulation*. 2016;134:1039-1051.
7. Binder CJ, Horkko S, Dewan A, Chang MK, Kieu EP, Goodyear CS, Shaw PX, Palinski W, Witztum JL, Silverman GJ. Pneumococcal vaccination decreases atherosclerotic lesion formation: molecular mimicry between Streptococcus pneumoniae and oxidized LDL. *Nat Med*. 2003;9:736-743.
8. Chou MY, Fogelstrand L, Hartvigsen K, Hansen LF, Woelkers D, Shaw PX, Choi J, Perkmann T, Backhed F, Miller YI, Horkko S, Corr M, Witztum JL, Binder CJ. Oxidation-specific epitopes are dominant targets of innate natural antibodies in mice and humans. *J Clin Invest*. 2009;119:1335-1349.

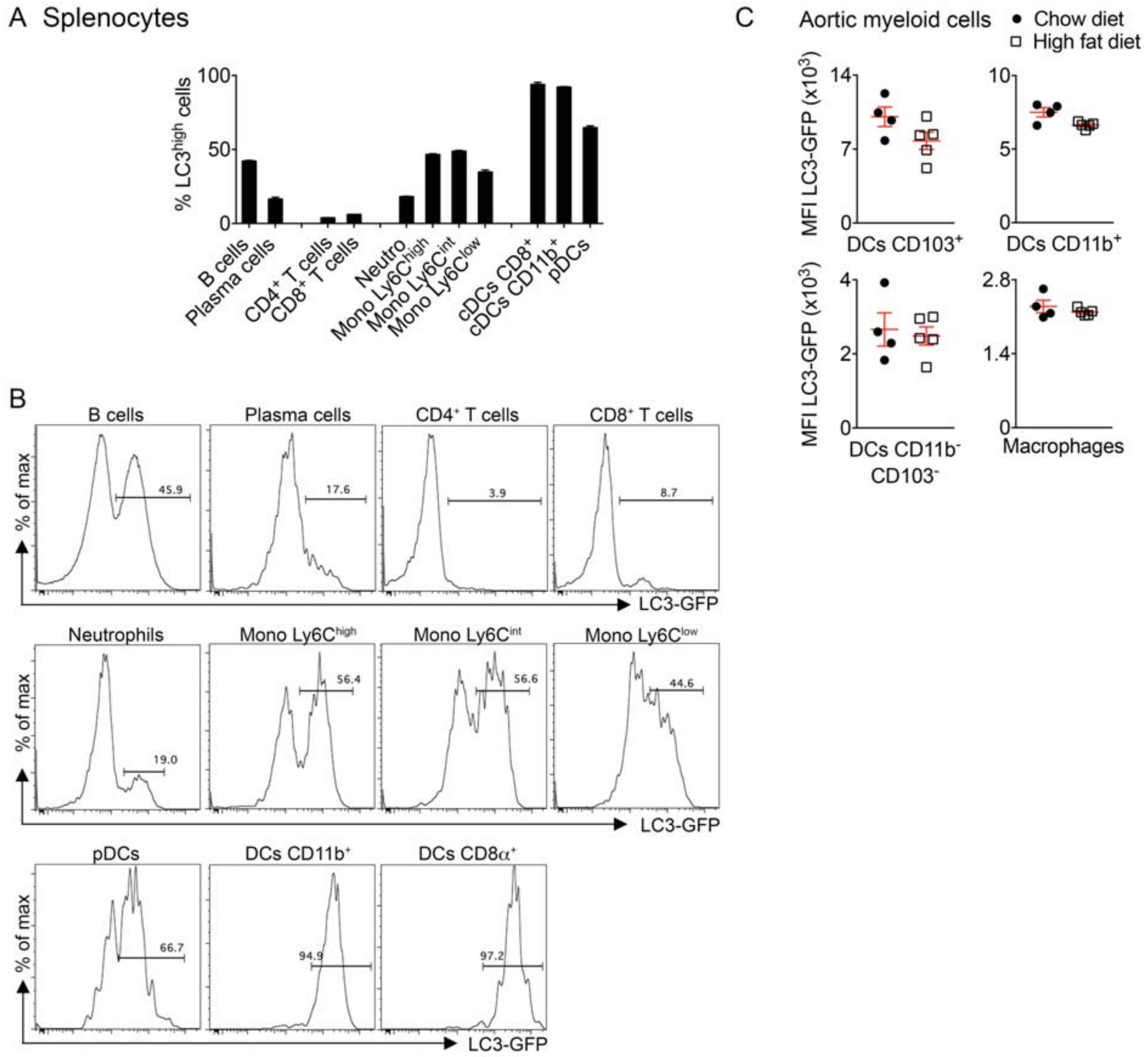

Online Figure I: LC3-GFP expression in splenocytes and aortic myeloid cells of *Ldlr$^{-/-}$* mice transplanted with LC3-GFP bone marrow.

A- Quantification of LC3-GFP^{+} cells in the different immune cell subsets.

B- Representative flow histograms showing the expression of LC3-GFP in each immune cell subset from the spleen.

C- Quantification of LC3-GFP expression in aortic cell suspension obtained from *Ldlr$^{-/-}$* female mice fed a chow or HFD for 8 weeks after bone marrow transplantation from LC3-GFP transgenic mice.

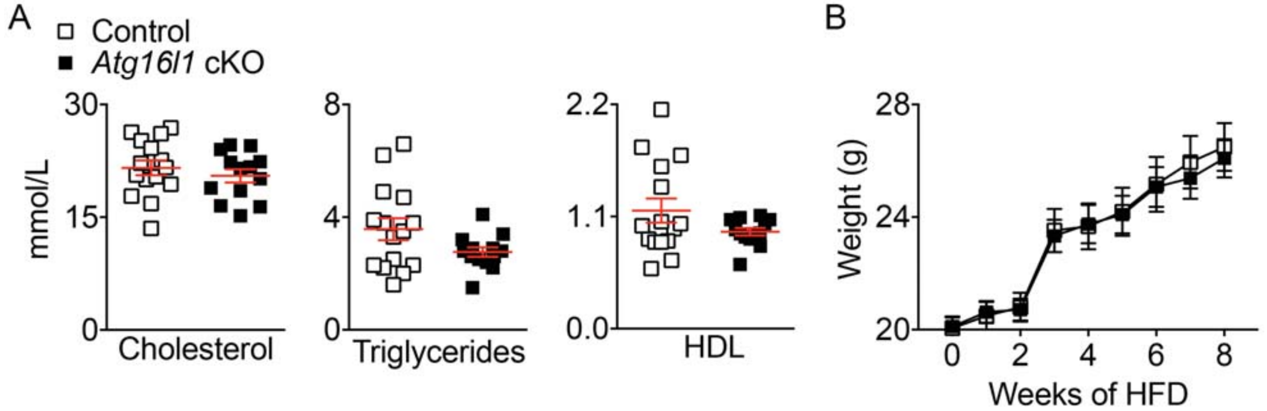

Online Figure II. Plasma lipid profiles and animal weight are not altered by *Atg16l1* deficiency in CD11c expressing cells.
A, B- Lipid profiles in the plasma (A) and animal weight (B) of *Ldlr*$^{-/-}$ mice transplanted with Control or *Atg16l1* cKO bone marrow and fed a high fat diet for 8 weeks. Data are representative of 2 independent experiments.

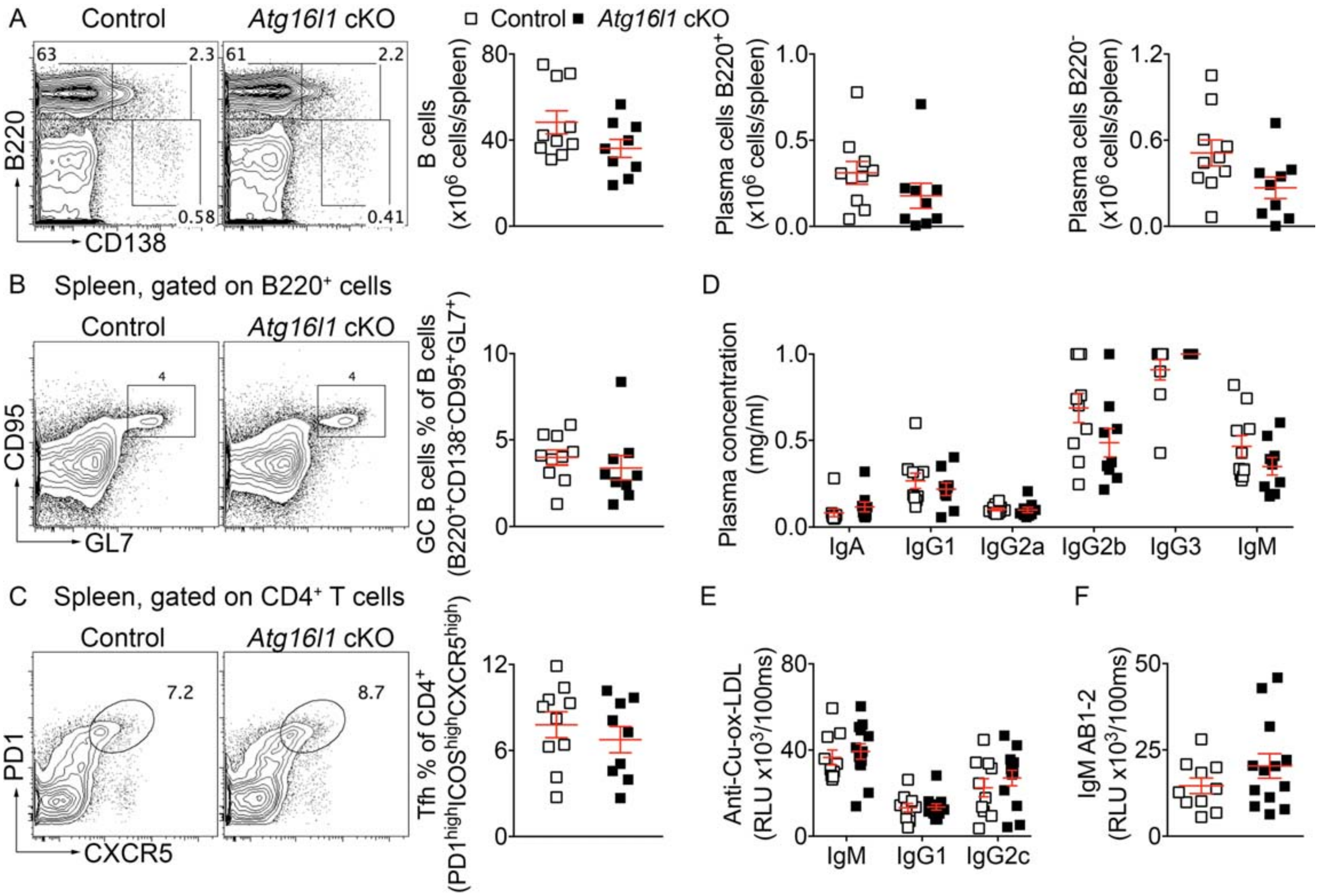

Online Figure III. Humoral immune response to high fat diet is not affected by *Atg16l1* deficiency in CD11c expressing cells.

A-C- Representative flow charts and quantification of mature B cells (B220^{+}CD138^{-}), plasmablasts (B220^{+}CD138^{+}) and plasma cells (B220^{-}CD138^{+}) (A), germinal center B cells (B220^{+}CD138^{-}CD95^{+}GL7^{+}) (B) and T follicular helper cells (Tfh, CD3^{+}CD4^{+}PD1^{high}ICOS^{high}CXCR5^{high}) in the spleens of *Ldlr*$^{-/-}$ mice transplanted with control (n=10) and *Atg16l1* cKO (n=9) bone marrow and put on high fat diet for 8 weeks.

D- Multiplex analysis of circulating antibody isotypes in the plasma of *Ldlr*$^{-/-}$ mice transplanted with control (n=10) and *Atg16l1* cKO (n=9) bone marrow and put on HFD for 8 weeks.

E, F- Quantification of IgM, IgG1 and IgG2c anti-Cu-ox-LDL (E) and natural IgM anti-PC (F) in the plasma of *Ldlr*$^{-/-}$ mice transplanted with control (n=10) and *Atg16l1* cKO (n=13) bone marrow and put on HFD for 8 weeks.

A-F: Data are representative of 2 independent experiments.

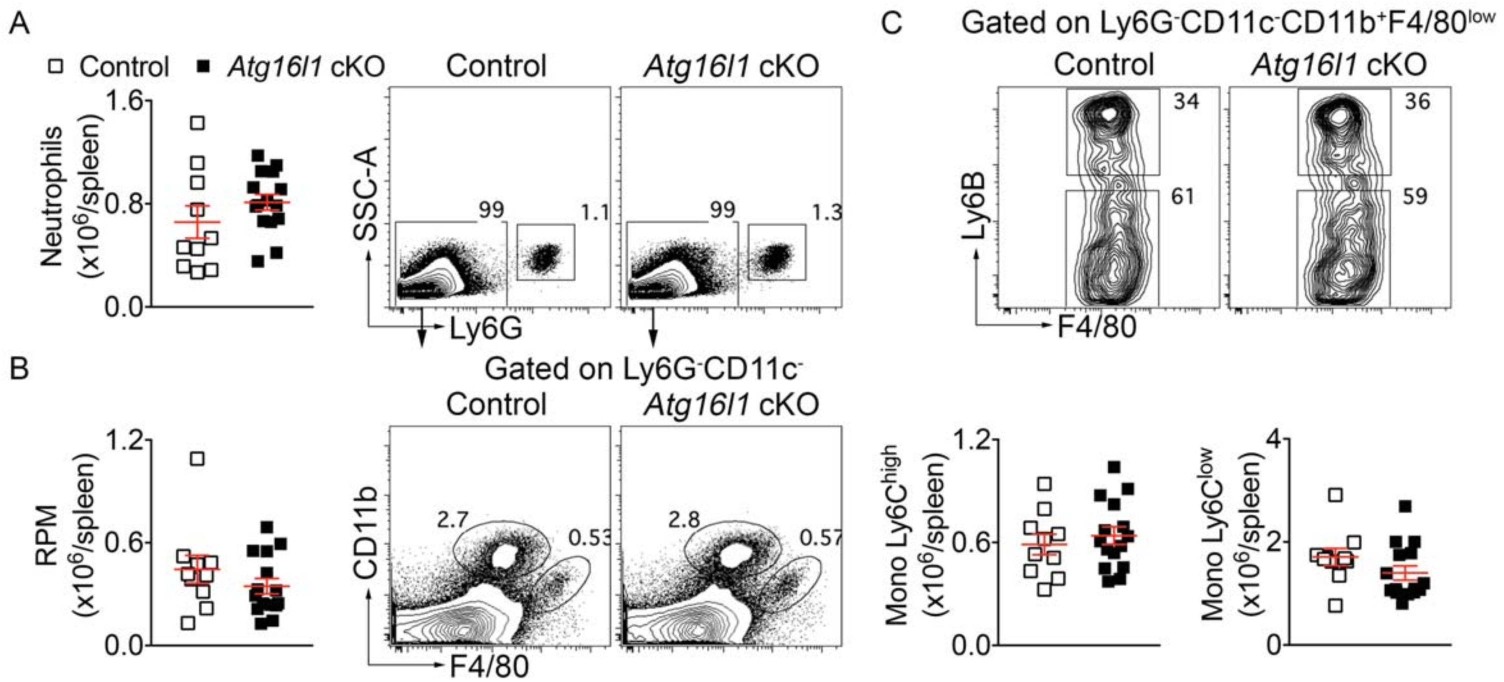

Online Figure IV. Splenic myeloid cell numbers are not affected by *Atg16l1* deficiency in CD11c expressing cells.

A-C- Quantification and representative flow charts of neutrophils (A), red pulp macrophages (RPM, B) and monocytes (C) in the spleens of *Ldlr*$^{-/-}$ mice transplanted with control (n=10) and *Atg16l1* cKO (n=15) bone marrow and put on high fat diet for 8 weeks.

A-C: Data are representative of 2 independent experiments.

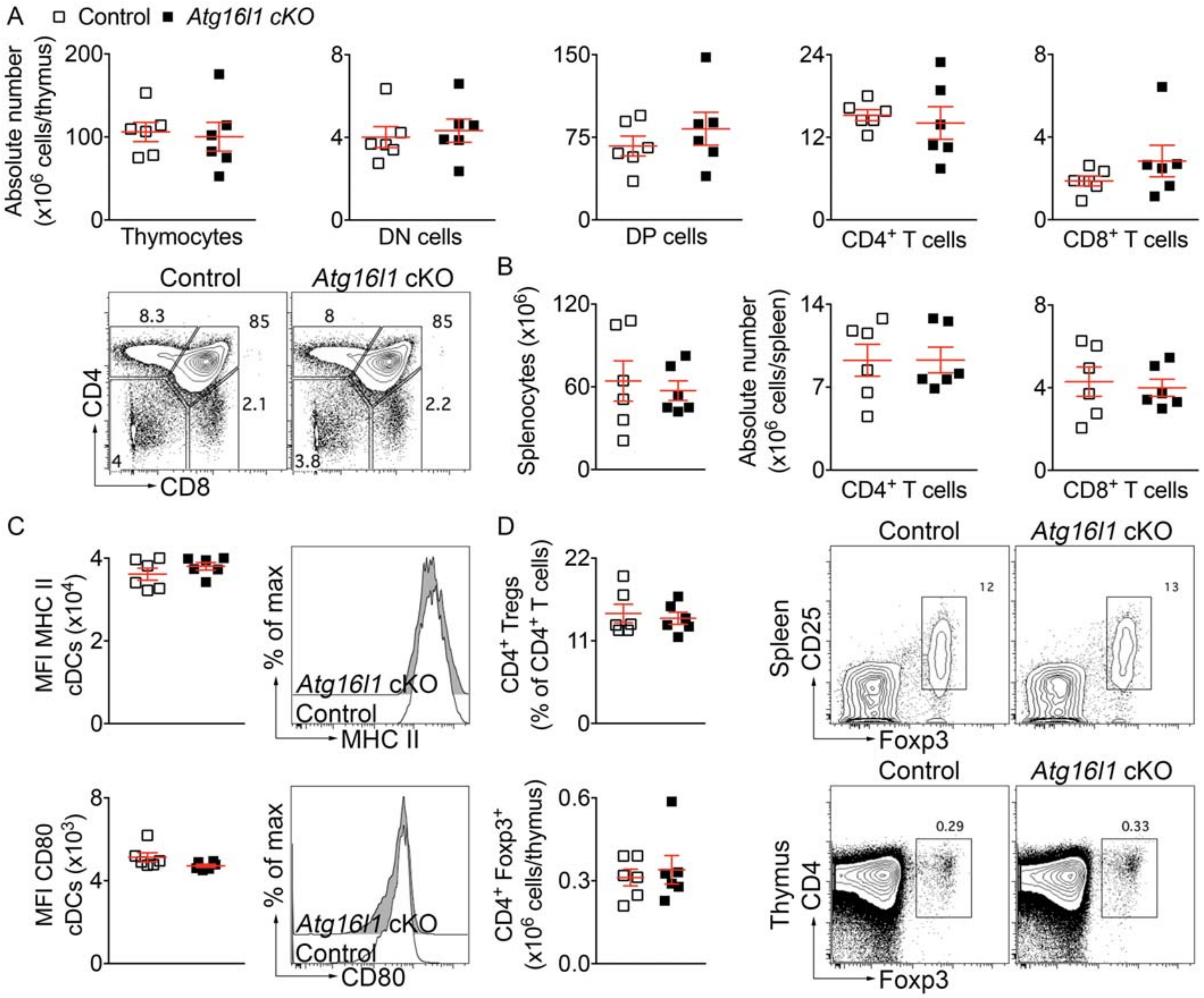

Online Figure V. *Atg16l1* deficiency in CD11c expressing cells does not affect immune cell development under steady state condition.

C57Bl/6J mice were transplanted with control (n=5) or *Atg16l1* cKO (n=5) bone marrow and the reconstitution of the immune system was analyzed after 4 weeks.

A, B- Quantification and representative flow charts of the different subsets of T cells in the thymus (A) and spleen (B).

C- Quantification of the expression of MHC II and CD80 by conventional dendritic cells (cDCs) and representative flow charts.

D- Quantification and representative flow charts of $CD4^+$ Tregs in the spleen and thymus.

A-D: Data are from one experiment.

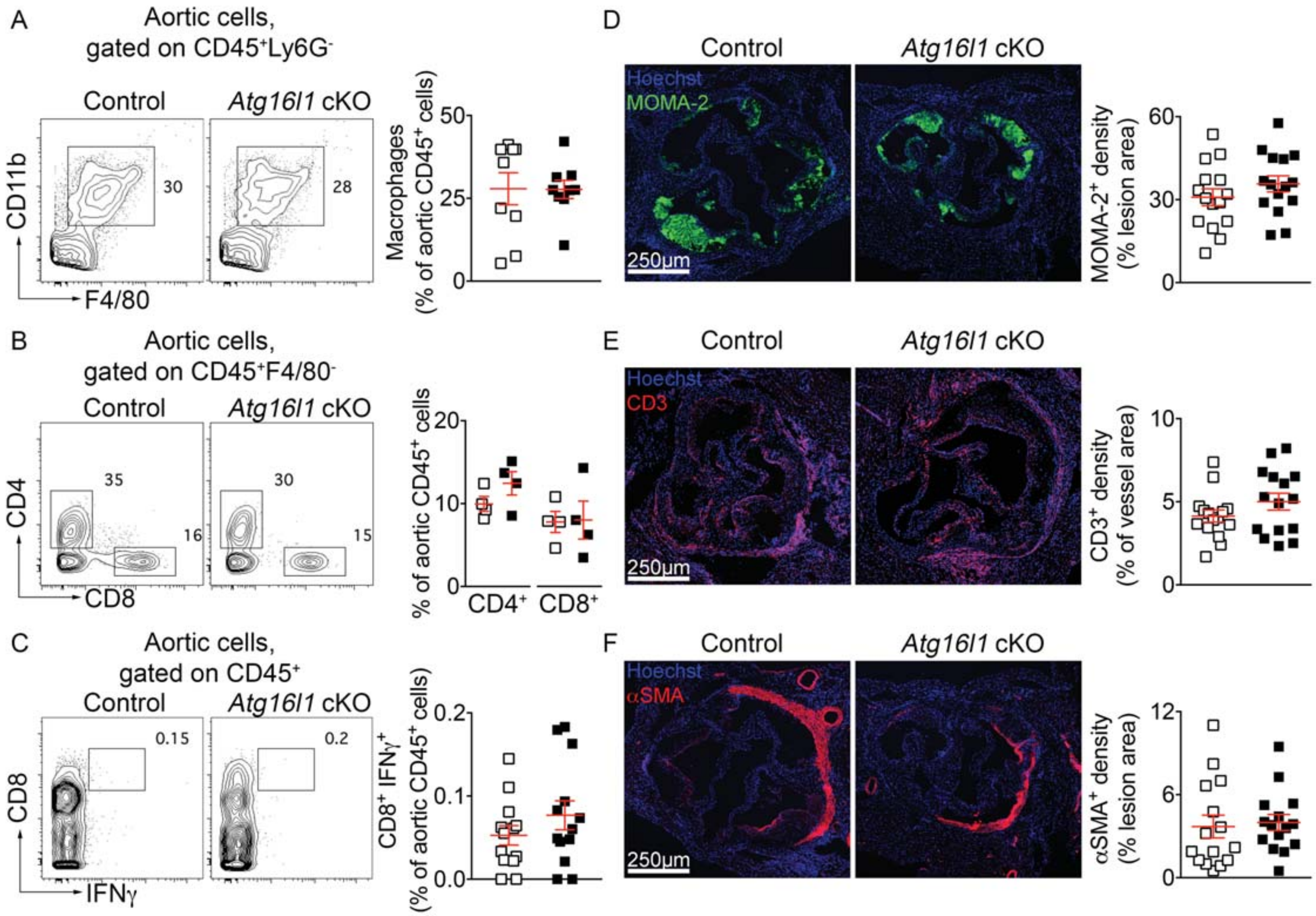

Online Figure VI. Atherosclerotic plaque composition of *Ldlr$^{-/-}$* mice with or without *Atg16l1* deficiency in CD11c expressing cells.

A-C- Representative flow charts and quantification of macrophages (A), $CD4^+$ and $CD8^+$ T cells (B), and IFNγ producing $CD8^+$ T cells (C) in the aorta of *Ldlr$^{-/-}$* mice transplanted with control (n=4-9) and *Atg16l1* cKO (n=4-13) bone marrow and put on high fat diet for 8 weeks.

D-F Representative images and quantification of foam cell formation (MOMA-2, D), T cell (CD3, E), and smooth muscle cell (αSMA, F) accumulation in lesions using immunofluorescent microscopy on cross sections of aortic sinus from *Ldlr$^{-/-}$* mice transplanted with control (n=15) and *Atg16l1* cKO (n=15) bone marrow and put on HFD for 8 weeks.

A- Graph is made with pooled data from 2 independent experiments.

B- Graph is made with data from one experiment.

C- Graph is made with pooled data from 3 independent experiments.

D-F- Representative data from 2 independent experiments.

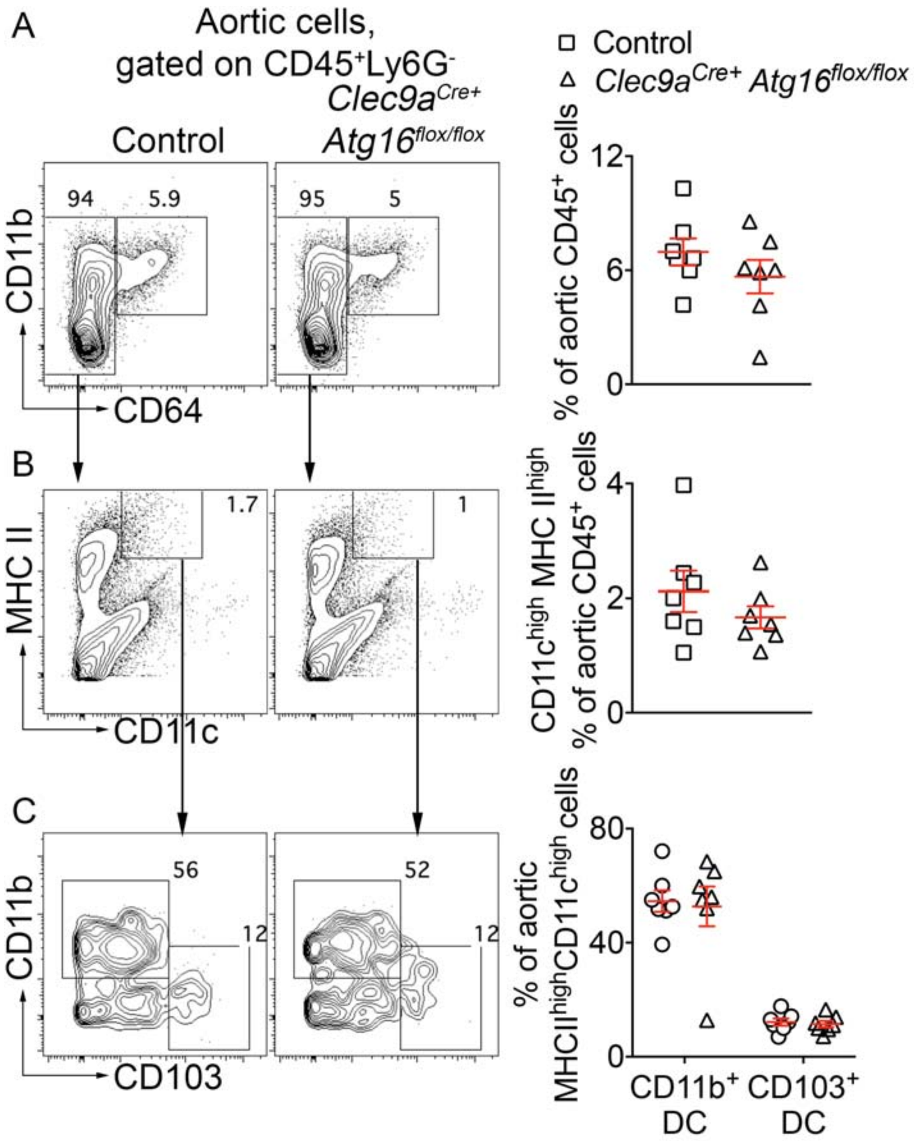

Online Figure VII. ***Atg16l1*** **deficiency in dendritic cells (DCs) derived from a Clec9a^{+} progenitor does not affect macrophage and DC accumulation in the aorta of** ***Ldlr$^{-/-}$*** **mice.**

A-C- Representative flow charts and quantification of macrophages (A), DCs (B), CD11b^{+} and CD103^{+} DCs (C) in the aortas of *Ldlr$^{-/-}$* mice transplanted with control (n=7) and *Clec9a^{Cre+} Atg16l1$^{flox/flox}$* (n=7) bone marrow and put on HFD for 8 weeks. Data were obtained from one experiment.

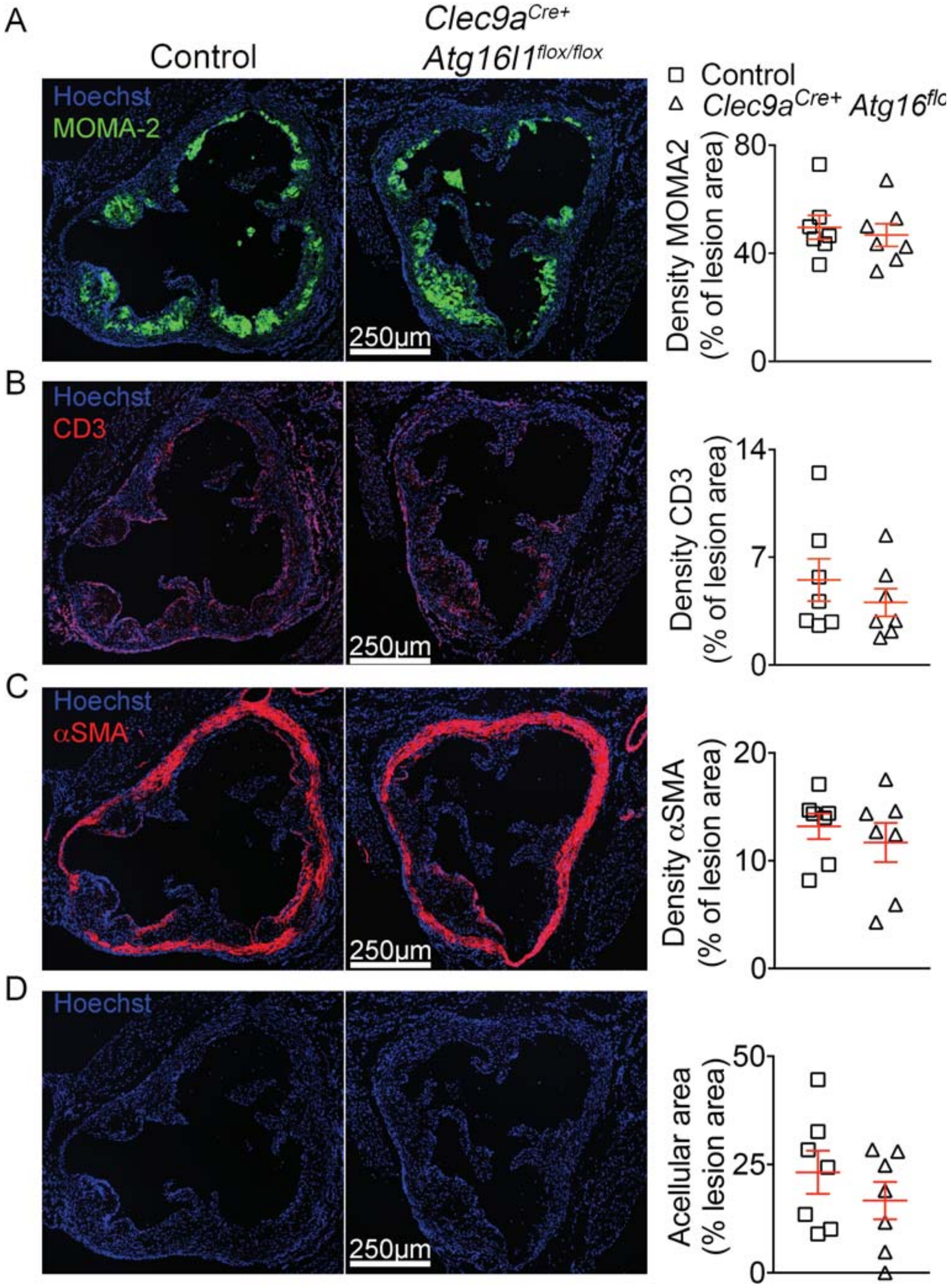

Online Figure VIII. Atherosclerotic plaque composition of *Ldlr$^{-/-}$* mice with or without *Atg16l1* deficiency in dendritic cells (DCs) derived from a Clec9a^{+} progenitor.

A-D- Representative images and quantification of foam cell (MOMA2, A), T cell (CD3, B), and smooth muscle cell (αSMA, C) accumulation, as well as necrotic core size (acellular area, D) in atherosclerotic lesions, analyzed by immunofluorescent microscopy on cross sections of aortic sinus from *Ldlr$^{-/-}$* mice transplanted with control (n=7) and *Clec9a^{Cre+} Atg16l1$^{flox/flox}$* (n=7) bone marrow and put on high fat diet for 8 weeks. Data are representative of 2 independent experiments.

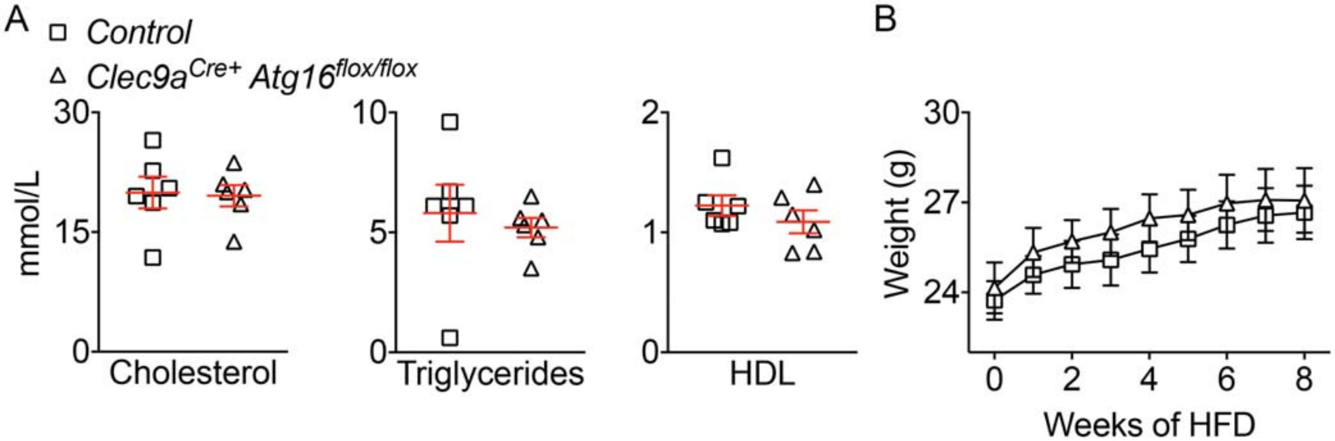

Online Figure IX. Plasma lipid profiles and animal weight are not altered by *Atg16l1* deficiency in dendritic cells derived from a Clec9a^{+} progenitor.
A, B- Lipid profile in the plasma (A) and animal weight (B) of *Ldlr*$^{-/-}$ mice transplanted with control (n=6) and *Clec9a*$^{Cre+}$ *Atg16l1*$^{flox/flox}$ (n=6) bone marrow and fed a HFD for 8 weeks.
Data are representative of 2 independent experiments.

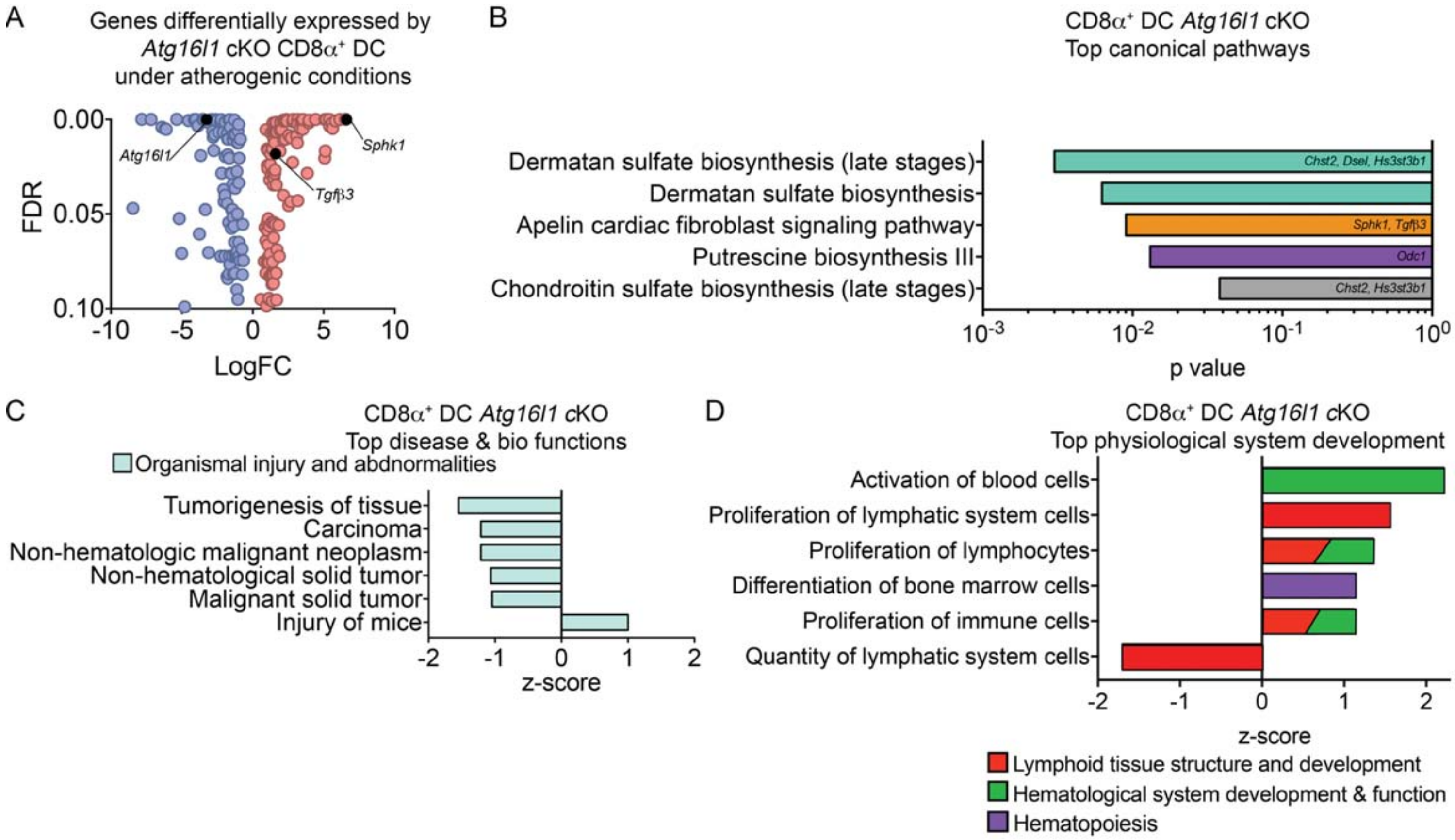

Online Figure X. Transcriptomic analysis of CD8α+ dendritic cells from *Ldlr-/-* mice transplanted with control or *Atg16l1* cKO bone marrow and put on high fat diet for 8 weeks.

A- Differentially expressed genes by *Atg16l1* cKO CD8α+ DCs (normalized on Control CD8α+ DCs; FDR<0.1, p<0.002) from *Ldlr-/-* mice transplanted with control (n=3) or *Atg16l1* cKO (n=2) bone marrow and put on HFD for 8 weeks.

B-D: Ingenuity analysis of the differentially expressed gene involvement in the top canonical pathways (B), Top disease and bio-functions (C) and Top physiological system development (D).

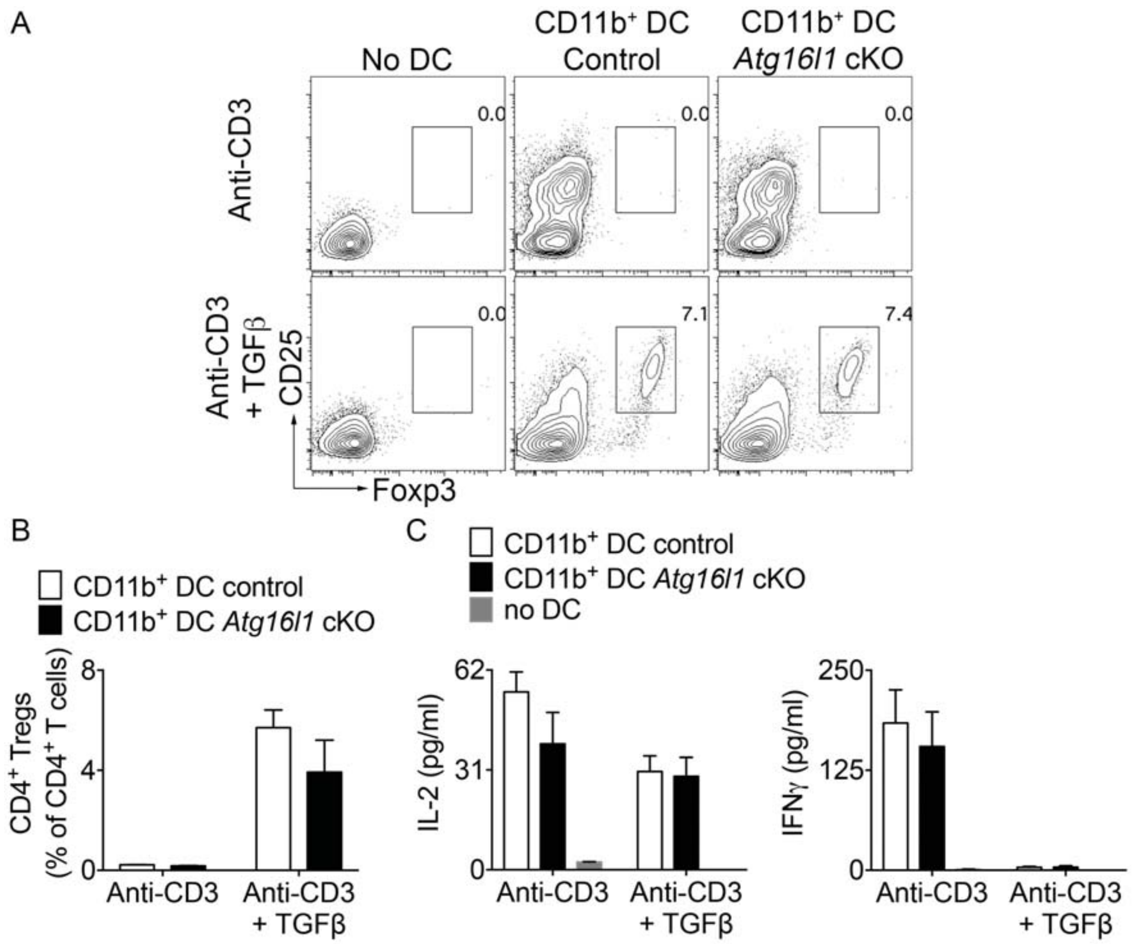

Online Figure XI. ***Atg16l1*** **deficient CD11b$^+$ DC do not promote CD4$^+$ Treg polarization under antigen non-specific conditions.**

A, B- Representative flow chart (A) and quantification (B) of CD4$^+$ Tregs (Foxp3$^+$CD25^{high}) generated from naïve CD4$^+$ OTII cells after co-culture with FACSpurified CD11b$^+$ DCs from control or *Atg16l1* cKO mice in the presence of soluble anti-CD3, with or without TGFβ.

C- Quantification of IL2 and IFNγ secretion in the supernatants of DC-OTII co-cultures described in A and B.

Data are representative of 2 independent experiments.

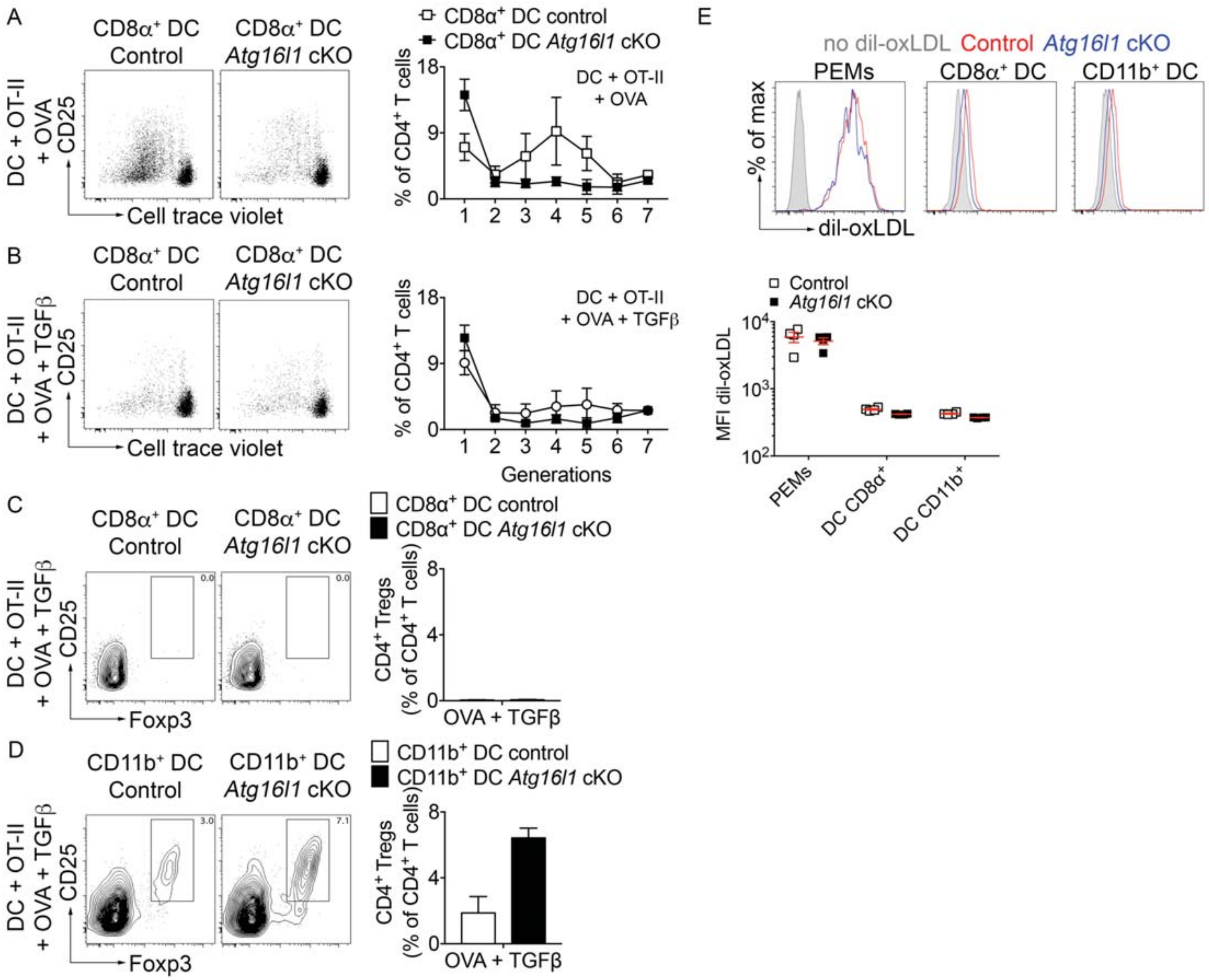

Online Figure XII. ***Atg16l1*** **deficient CD8α⁺ DC do not promote antigen specific CD4⁺ Treg polarization.**

A, B- Representative flow chart of cell trace violet dilution and quantification of the proliferation of FACS-purified naïve $CD4^+$ OTII cells co-cultured with FACS-purified $CD8\alpha^+$ DC and OVA protein, without (A) or with TGFβ (B).

C, D- Representative flow chart and quantification of the proportion of $CD4^+$ Tregs ($Foxp3^+CD25^{high}$) in cocultures of FACS-purified naïve OTII $CD4^+$ T cells and $CD8\alpha^+$ (C) or $CD11b^+$ (D) DCs from control or *Atg16l1* cKO mice in the presence of OVA protein and TGFβ. Data were obtained using technical triplicates and are representative of 2 independent experiments.

E- Representative flow chart showing the uptake of dil-oxLDL (1 µg/ml) ex-vivo by peritoneal macrophages (PEMs) or splenic dendritic cell subsets for 16 hours.

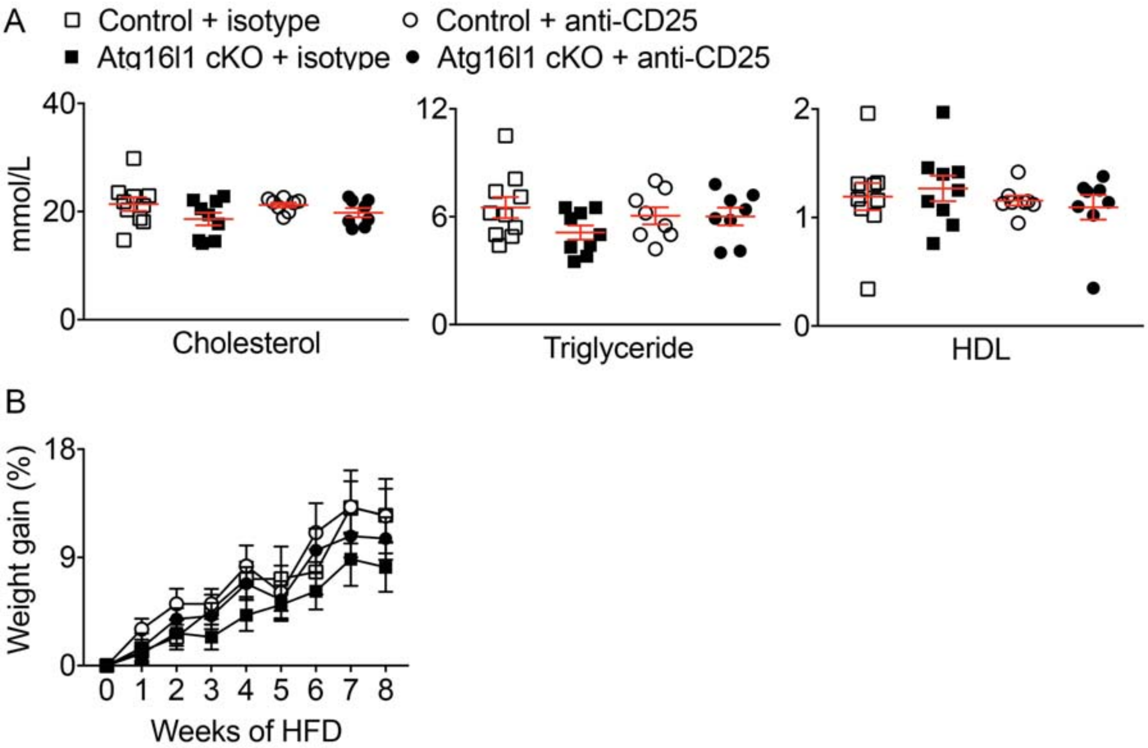

Online Figure XIII. Plasma lipid profiles and animal weights in *Ldlr*$^{-/-}$ mice transplanted with control or *Atg16l1* cKO bone marrow, treated with isotype-matched or anti-CD25 antibody, and fed a high fat diet for 8 weeks.

A, B- Lipid profile in the plasma (A) and weight gain (B); Control + isotype, n=10; *Atg16l1* cKO + isotype, n=8; Control + anti-CD25, n=8; *Atg16l1* cKO + anti-CD25, n=8. Data were obtained from one experiment.

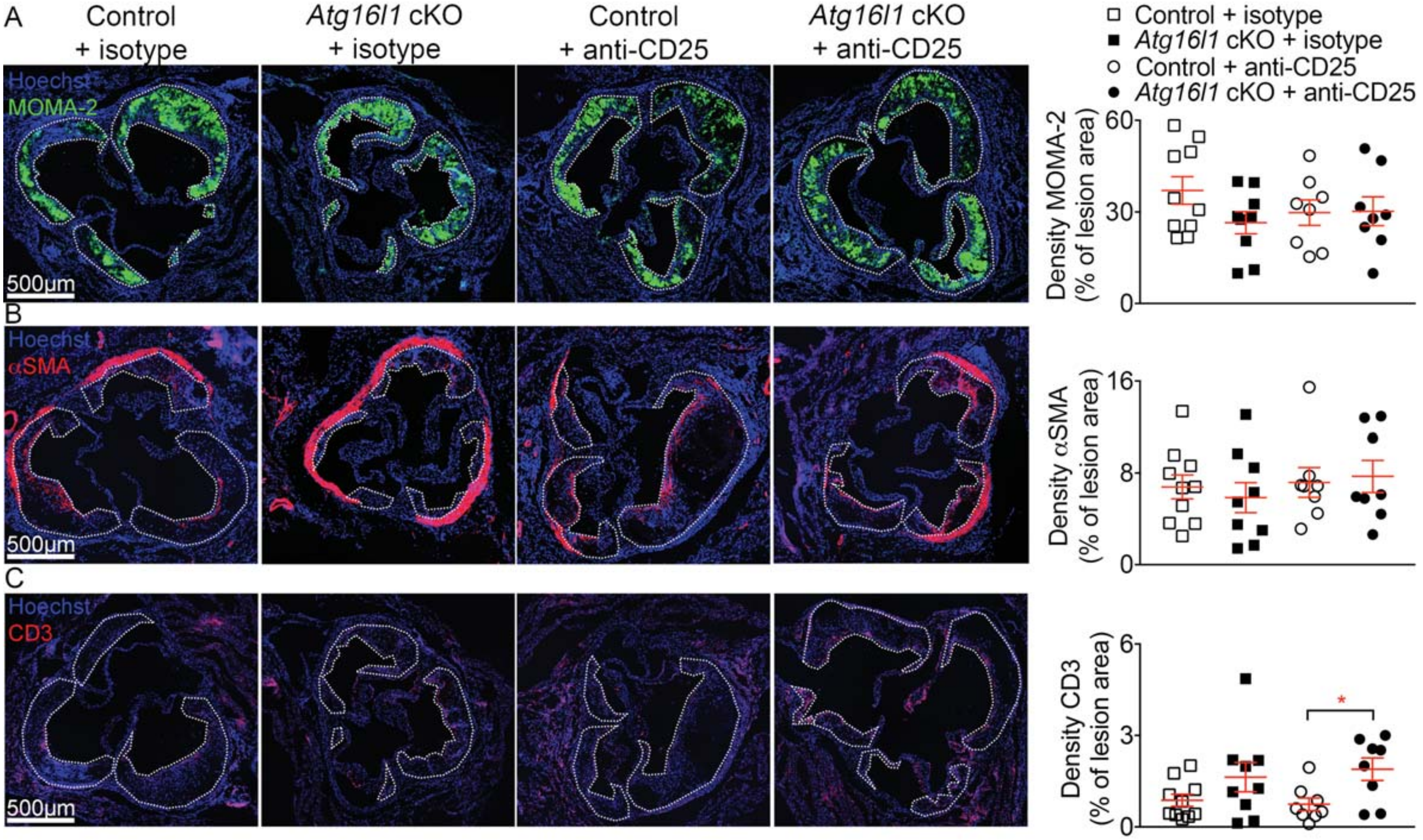

Online Figure XIV. Anti-CD25 (PC61) treatment abrogates the atheroprotective effect of *Atg16l1* deficiency in CD11c expressing cells.
A-C Representative images and quantification of foam cell (MOMA-2, A), smooth muscle cell (αSMA, B), and T cell (CD3, C) accumulation in lesions using immunofluorescent microscopy on cross sections of aortic sinus from *Ldlr*$^{-/-}$ mice transplanted with control or *Atg16l1* cKO bone marrow, injected with isotype-matched or anti-CD25 antibody and put on high fat diet for 8 weeks. Control + isotype, n=10; *Atg16l1* cKO + isotype, n=8; Control + anti-CD25, n=8; *Atg16l1* cKO + anti-CD25, n=8. *p<0.05 Control + anti-CD25 vs *Atg16l1* cKO + anti-CD25, Kruskal-Wallis test and uncorrected Dunn’s post-test. Data were obtained from one experiment.

Online Table I. Differentially expressed genes in DCs from *Atg16l1* cKO mice.
See Excel file.

Panel aortic dendritic cells				
**Antigen**	**Fluorochome**	**Clone**	**Manufacturer**	**cat number**
CD45	eVolve™ 605	30-F11	Thermofisher	63-0451-80
MHC Class II (I-A/I-E)	eFluor® 450	M5/114.15.2	Thermofisher	48-5321-82
CD11c	PE/Cy7	N418	Biolegend	117317
CD11b	Alexa Fluor® 700	M1/70	BD Biosciences	557960
CD103	FITC	2E7	Thermofisher	11-1031-82
CD64	APC-Vio770	REA286	Miltenyi Biotec	130-118-685
F4/80	Alexa Fluor® 647	CI:A3-1	Bio-Rad	MCA497A647T
Ly6G	PE	1A8	BD Biosciences	561104

Panel T cells				
**Antigen**	**Fluorochome**	**Clone**	**Manufacturer**	**cat number**
CD3	Alexa Fluor® 488	145-2C11	Biolegend	100321
CD4	Alexa Fluor® 700	RM4-5	Biolegend	100536
CD8α	Brilliant Violet 785™	53-6.7	Biolegend	100750
CD44	APC	IM7	Biolegend	103011

Panel CD4+ Tregs				
**Antigen**	**Fluorochome**	**Clone**	**Manufacturer**	**cat number**
CD3	Alexa Fluor® 488	145-2C11	Biolegend	100321
CD4	Alexa Fluor® 700	RM4-5	Biolegend	100536
CD25	APC	PC61	Biolegend	102011
Foxp3	PE	FJK-16s	Thermofisher	12-5773-82

Panel Tfh				
**Antigen**	**Fluorochome**	**Clone**	**Manufacturer**	**cat number**
CD3	Alexa Fluor® 488	145-2C11	Biolegend	100321
CD4	Alexa Fluor® 700	RM4-5	Biolegend	100536
ICOS	APC	C398.4A	Thermofisher	17-9949-82
PD1	PE/Cy7	RMP1-30	Biolegend	109110
CXCR5	PE	L138D7	Biolegend	145503

Panel Tfh				
**Antigen**	**Fluorochome**	**Clone**	**Manufacturer**	**cat number**
B220	Brilliant Violet 605™	RA3-6B2	Biolegend	103243
CD138	APC	281-2	Biolegend	142505
GL7	eFluor® 450	GL7	Thermofisher	48-5902-82
CD95	PE/Cy7	Jo2	BD Biosciences	557653

Panel spleen myeloid cells				
**Antigen**	**Fluorochome**	**Clone**	**Manufacturer**	**cat number**
CD11c	PE	N418	Biolegend	117307
Ly6G	V450	1A8	BD Biosciences	560603
Ly6B	Alexa Fluor® 647	7/4	Bio-Rad	MCA771A647T
CD11b	Alexa Fluor® 700	M1/70	BD Biosciences	557960
F4/80	PE-Cy7	BM8	Thermofisher	25-4801-82

Panel spleen myeloid cells				
**Antigen**	**Fluorochome**	**Clone**	**Manufacturer**	**cat number**
CD4	Alexa Fluor® 700	RM4-5	Biolegend	100536
CD8α	Brilliant Violet 785™	53-6.7	Biolegend	100750
IFNγ	APC	XMG1.2	BD Biosciences	562018

Online Table II: Antibodies used for flow cytometry

Antigen	Conjugated	Species, Clone	Manufacturer	Cat number
MHC Class II (I-A/I-E)	Unconjugated	Rat, M5/114.15.2	Thermofisher	14-5321-82
CD3	Unconjugated	Rabbit, polyclonal	DAKO	A0542
MOMA-2	Unconjugated	Rat, MOMA-2	Bio-rad	MCA519GT
aSMA	Cy3	mouse, 1A4	Sigma	C6198
CD11c	Unconjugated	Hamster, N418	Biolegend	117302
LC3	Unconjugated	Rabbit, D11	Cell Signaling	3868S
Rat IgG	Alexa fluor 488	Goat, polyclonal	Thermofisher	A-11006
Rabbit IgG	Alexa fluor 555	Goat, polyclonal	Thermofisher	A-21428
Rabbit IgG	Alexa fluor 647	Goat, polyclonal	Thermofisher	A-21245
Hamster IgG	Alexa fluor 568	Goat, polyclonal	Abcam	ab175716

Online Table III: Antibodies used for immunofluorescent microscopy
