# Supplementary figures and images for: Impaired Autophagy in CD11b+ Dendritic Cells Expands CD4+ Regulatory T Cells and Limits Atherosclerosis in Mice
Source: Circ Res. 2019 Nov 7;125(11):1019–34. doi: 10.1161/CIRCRESAHA.119.315248 (PMC6844650; doi:10.1161/CIRCRESAHA.119.315248)

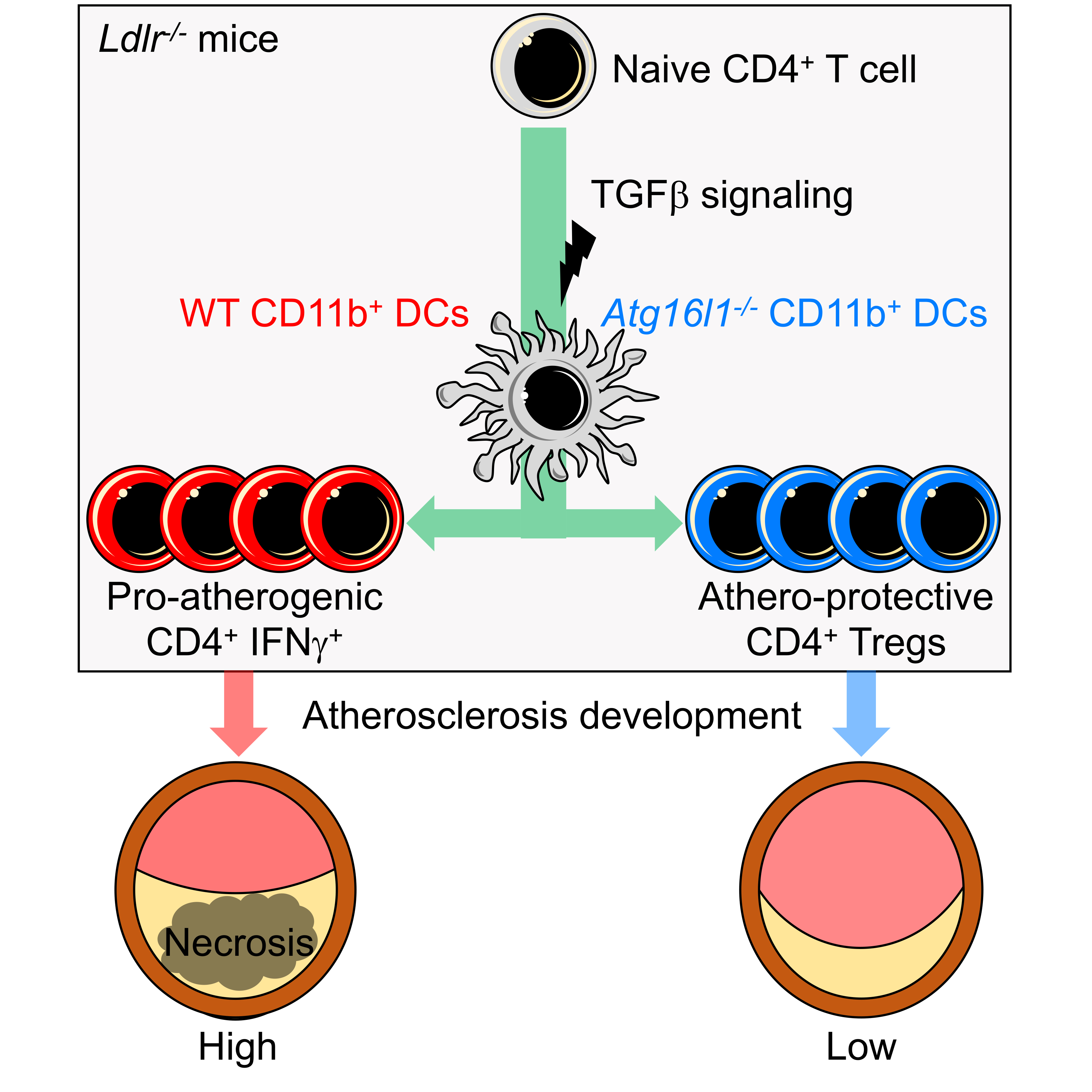

Supplement: Supplementary file 4 [file res-125-1019-s004.jpg]
